# Narrow Bandgap Metal Halide Perovskites for All-Perovskite Tandem Photovoltaics

**DOI:** 10.1021/acs.chemrev.3c00667

**Published:** 2024-03-25

**Authors:** Shuaifeng Hu, Jarla Thiesbrummel, Jorge Pascual, Martin Stolterfoht, Atsushi Wakamiya, Henry J. Snaith

**Affiliations:** a Clarendon Laboratory, Department of Physics, University of Oxford, Oxford OX1 3PU, United Kingdom; b Institute for Chemical Research, Kyoto University, Gokasho, Uji, Kyoto 611-0011, Japan; c Institute for Physics and Astronomy, University of Potsdam,14476 Potsdam-Golm, Germany; d Polymat, University of the Basque Country UPV/EHU, 20018 Donostia-San Sebastian, Spain; e Electronic Engineering Department, The Chinese University of Hong Kong, Hong Kong 999077, SAR China

## Abstract

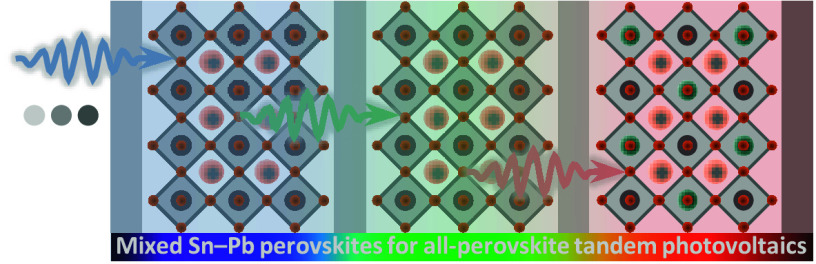

All-perovskite tandem solar cells are attracting considerable interest in photovoltaics research, owing to their potential to surpass the theoretical efficiency limit of single-junction cells, in a cost-effective sustainable manner. Thanks to the bandgap-bowing effect, mixed tin−lead (Sn−Pb) perovskites possess a close to ideal narrow bandgap for constructing tandem cells, matched with wide-bandgap neat lead-based counterparts. The performance of all-perovskite tandems, however, has yet to reach its efficiency potential. One of the main obstacles that need to be overcome is the—oftentimes—low quality of the mixed Sn−Pb perovskite films, largely caused by the facile oxidation of Sn(II) to Sn(IV), as well as the difficult-to-control film crystallization dynamics. Additional detrimental imperfections are introduced in the perovskite thin film, particularly at its vulnerable surfaces, including the top and bottom interfaces as well as the grain boundaries. Due to these issues, the resultant device performance is distinctly far lower than their theoretically achievable maximum efficiency. Robust modifications and improvements to the surfaces of mixed Sn−Pb perovskite films are therefore critical for the advancement of the field. This Review describes the origins of imperfections in thin films and covers efforts made so far toward reaching a better understanding of mixed Sn−Pb perovskites, in particular with respect to surface modifications that improved the efficiency and stability of the narrow bandgap solar cells. In addition, we also outline the important issues of integrating the narrow bandgap subcells for achieving reliable and efficient all-perovskite double- and multi-junction tandems. Future work should focus on the characterization and visualization of the specific surface defects, as well as tracking their evolution under different external stimuli, guiding in turn the processing for efficient and stable single-junction and tandem solar cell devices.

## 1 Introduction

Following the policies for the decarbonization of our energy system, the development of new-generation sustainable green energy is urgently needed.^13,14^ Metal halide perovskites are a new type of material, possessing exceptionally high adsorption coefficients, tunable bandgaps, and solution- and dry-processable fabrication protocols.^16−19^ Thus, they have enabled us to advance photovoltaic solar energy conversion efficiency and promise to deliver a low-cost photovoltaic (PV) technology that can be sustainable at the multiterawatt scale.^21−24^ The most efficient, and arguably closest to-market, type of perovskite-containing photovoltaics are two-terminal monolithic tandems, with perovskite-on-Si leading the efficiency and scale-up race, and perovskite−perovskite tandems in close pursuit.^22,27^ In these devices, the efficient utilization of solar energy is achieved through collectively harvesting a broad portion of the solar spectrum while minimizing thermalization losses, with the two absorbers having different bandgaps.^34^ The optimal gap for the front perovskite subcell is ∼1.65 to 1.70 and ∼1.80 to 1.85 eV for perovskite-on-Si and perovskite−perovskite tandems, respectively, while the counterpart for the narrow-bandgap (NBG) rear perovskite subcell is generally ∼1.25 eV, which is currently the narrowest achievable bandgap for efficient metal halide perovskite photovoltaics.^27,34,37^ In perovskite−perovskite (perovskite-on-Si) tandems, the photons with higher energy are absorbed by the wide-bandgap (WBG) front perovskite absorber, while those with lower energy are harvested by the rear NBG perovskite (Si) absorber. Then, a charge recombination layer (CRL) is employed to connect those two subcell units. In this way, the devices present a theoretical maximum achievable efficiency under standard AM1.5 100 mW cm^−2^ irradiance of about 45%,^38,39^ which pronouncedly surpasses radiative limits for the single-junction solar cells, at approximately 33%, based on the principle of detailed balance.^45^ For single-junction perovskite solar cells (PSCs), within only about one decade since their first discovery,^46−49^ efficiencies surpassed 25%,^52,53^ for the devices constructed with both n-i-p (regular)^54−59^ and now p-i-n (inverted)^60−63^ stackings, putting them on par with state-of-the-art crystalline silicon solar cells, at 26.8%.^64,65^ For monolithic perovskite-on-Si and perovskite−perovskite tandems, the current record efficiencies reported have surpassed 33%^52^ and 29%,^71^ respectively, surpassing the Shockley−Queisser (S-Q) theoretical efficiency limit of 33.7% for single-junction solar cells,^45^ with the leading efficiency of 33.9% achieved by perovskite-on-Si tandem PV technology.^76^

**Figure 1:**
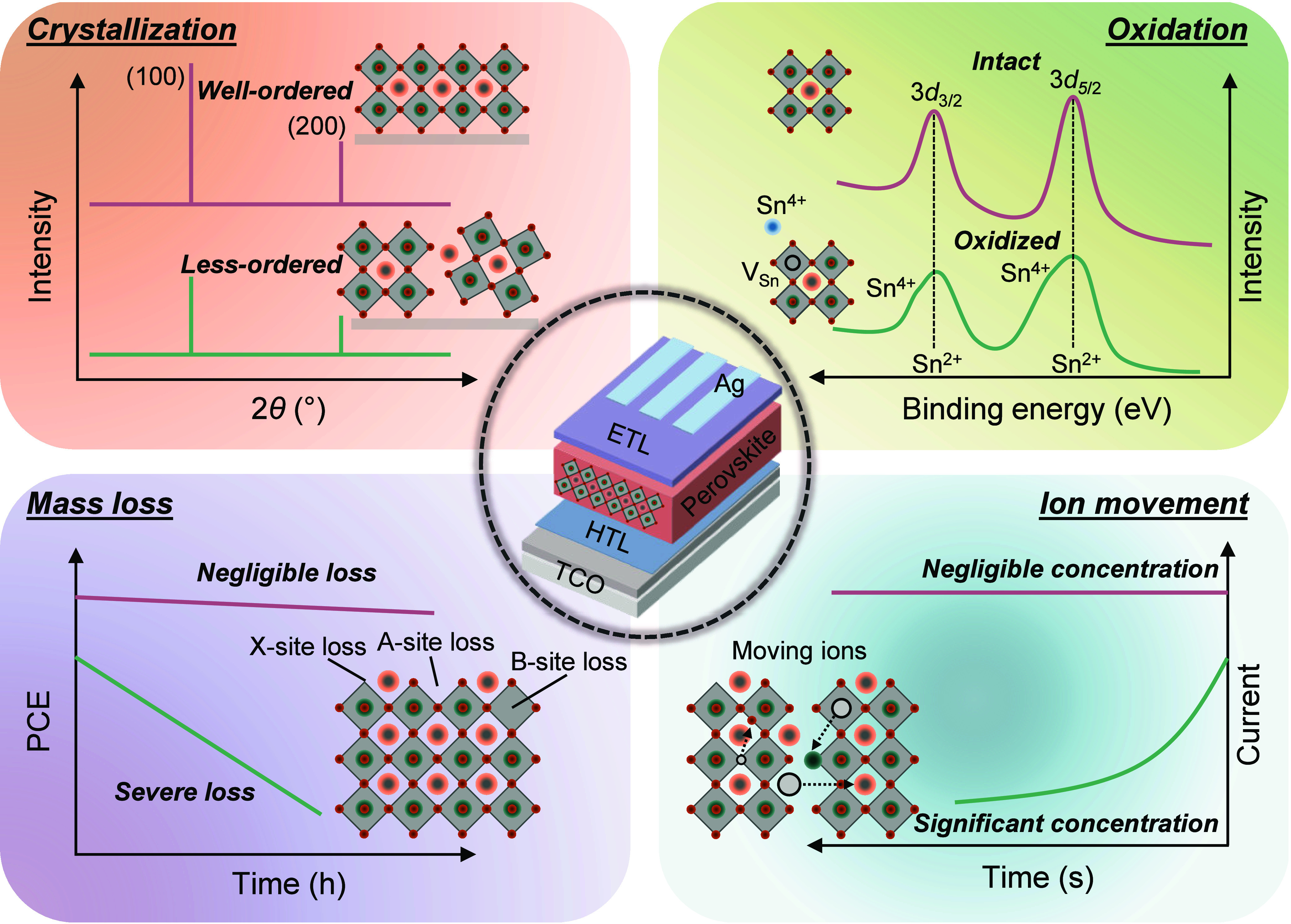
Origins of efficiency losses. Crystallization: Schematic illustration of well- and less-ordered crystallographic domains of perovskite films with the crystallinity reflected by XRD results. Oxidation: Sn(II) oxidizes to Sn(IV), leading to Sn(II) vacancies in the lattice and resulting in self-*p*-doping, with the extent of oxidation reflected by XPS results. Mass loss: The loss of perovskite lattice ions leads to a severe decline in cell performance. Ion movement: A significant concentration of the ions in perovskite films leads to an evident loss of current over time which leads to degradation and initial efficiency losses of the PSCs.

Considering the advantage of further lowering production costs, reducing embodied energy and energy payback, all-perovskite tandems including double- and multi-junction cells have been considered as a central target to be realized. Currently, one of the core limitations is on the NBG cell, which generally employs mixed Sn−Pb perovskites containing—most of the time—a half Sn(II) and half Pb(II) composition at the B-site of the 3D perovskite lattice. Unfortunately, the introduction of tin into the perovskite lattice comes with several emerging drawbacks, such as the facile oxidation of Sn(II),^78,79^ especially at the film surface,^12^ and the substandard quality of the films resulting from the fast and difficult to control crystallization dynamics.^85,86^ The consequent structural imperfections, i.e., vacancies, interstitial and substitutional sites, crystallographic phase impurities, reactive remnants, and lattice stacking faults, are dominantly located at the film surfaces,^87−89^ including interfaces and grain boundaries. Therefore, for mixed Sn−Pb PSCs, modifying the surfaces of the perovskite films is pivotal to improving their efficiency and durability.

In this Review, we first summarize different sources of efficiency losses for the mixed Sn−Pb PSCs, such as Sn(II) oxidation, unregulated crystallization, operational stress, and ion migration. We highlight particularly the impact of these defects at perovskite surfaces and the resulting severe nonradiative charge carrier recombination. The detailed surface states of perovskite films are discussed in the context of their structural, electronic, and defect characteristics. We discuss the strategies that have been employed at the different surfaces (top and bottom interfaces, grain boundaries) to mitigate these detrimental effects in single-junction and all-perovskite tandem devices, with specific highlights of the two-dimensional (2D) capping strategies. We then outline the important aspects of routinely and reproducibly integrating the NBG absorber into all-perovskite double- and multi-junction tandem devices. In the end, we discuss the future potential of surface characterization and modification for the mixed Sn−Pb perovskites from the view of intrinsic material properties and photovoltaic applications.

## 2 Origins of Efficiency Losses

To gain a global picture of the PSCs, understanding the origin of their efficiency losses is of particular importance. These losses are largely influenced by imperfections in the thin film generated during its fabrication, as well as due to their intrinsic instability. In this section, we will be discussing the importance of the crystallization process for the production of high-quality thin films, as well as the impact of oxidation, mass loss, and ion movement on the generation of defects affecting the device performance (Figure 1).

### 2.1 Crystallization

**Figure 2:**
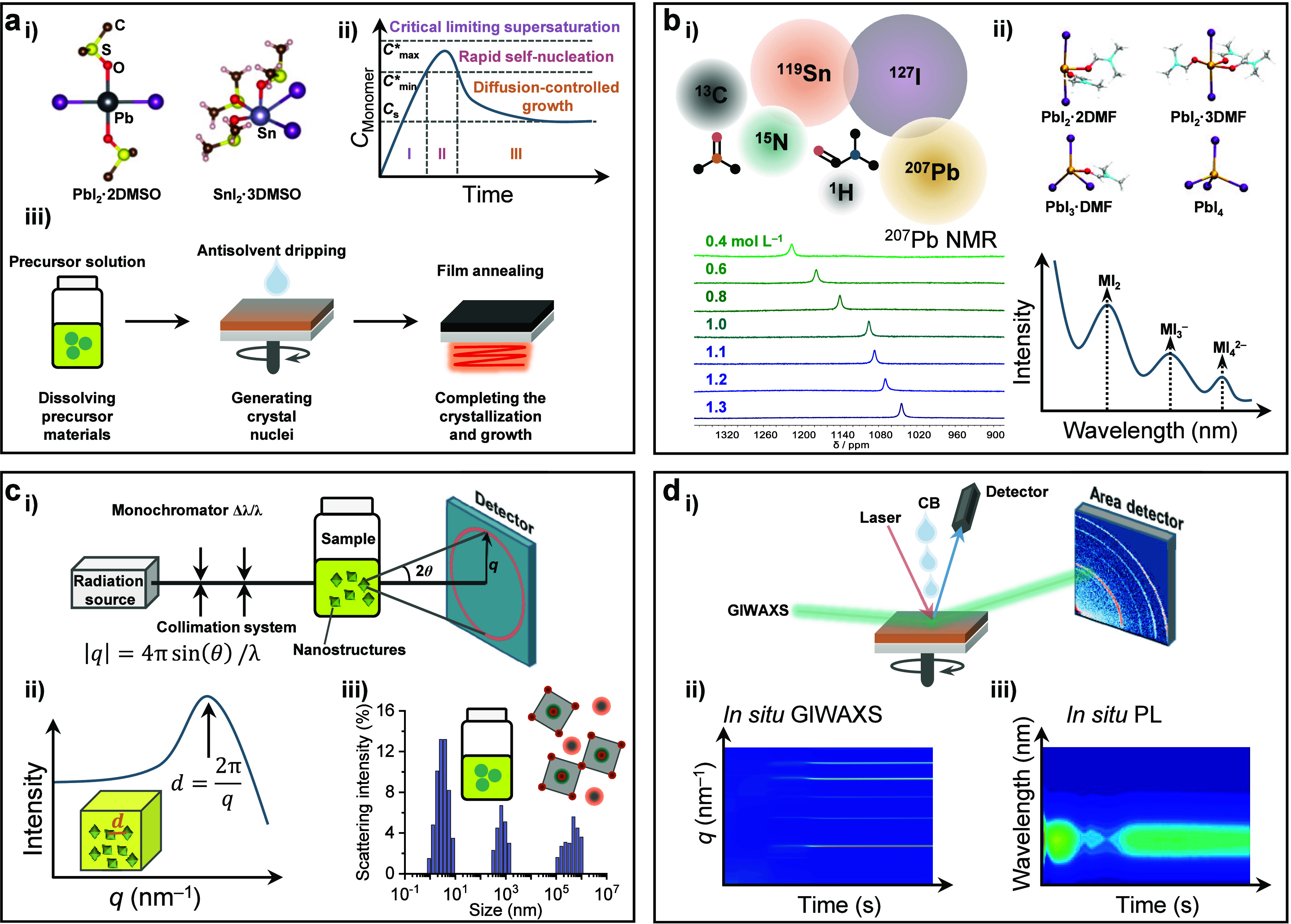
Crystallization. (a) (i) Crystal structure of PbI_2_·2DMSO and SnI_2_·3DMSO complexes generated with the CIF file from refs (4) and ref (6), respectively. (ii) La Mer diagram for monodispersed particle formation (homogeneous nucleation). *C*_S_ is the solubility, *C*_min_* is the minimum concentration for nucleation, i.e., the minimum supersaturation level for homogeneous nucleation, and *C*_max_* is the maximum concentration for nucleation. Regions I, II, and III represent prenucleation, nucleation, and growth stages, respectively. (iii) Processing of the spin-coated perovskite films with the main role of each process noted at the bottom. (b) (i) Isotopes of elements from the perovskite precursor materials active in NMR and ^207^Pb NMR spectra of the perovskite precursor solution with different concentrations. Reproduced with permission from ref (26). Copyright 2021 Royal Society of Chemistry under a Creative Commons Attribution 3.0 Unported License. (ii) Optimized geometries for selected [PbI_m_X_n_]^2−m^ iodoplumbate complexes in DMF solvent with the UV−vis absorption spectra giving the characteristic absorption of the iodoplumbates. Reproduced with permission from ref (28). Copyright 2019 American Chemical Society. (c) (i) Basic scheme of a small angle scattering instrument. Reproduced with permission from ref (26). Copyright 2021 Royal Society of Chemistry under a Creative Commons Attribution 3.0 Unported License. (ii) Representative SAXS pattern of the perovskite solution. (iii) DLS results of a mixed Sn−Pb perovskite precursor solution. Reproduced with permission from ref (9). Copyright 2022 Royal Society of Chemistry. (d) (i) Schematic illustration of *in situ* GIWAXS and PL measurements during the spin coating stage of perovskite film formation. Reproduced with permission from ref (50). Copyright 2022 American Chemical Society. (ii) Illustration of the *in situ* GIWAXS results. (iii) Illustration of the *in situ* PL results, taken from ref (67). Copyright 2021 American Association for the Advancement of Science under a Creative Commons Attribution License 4.0 (CC BY).

In the fabrication of solution-processed perovskite thin films, the final material will be polycrystalline with a large portion of surfaces (e.g., grain boundaries) and the consequent surface and structural defects, which can introduce trap states.^91,92^ Furthermore, defects will be present throughout the crystalline domains, and the density and prevalence of specific defects will be influenced by nucleation and growth conditions and environment. Hence, the crystallization process profoundly determines the final quality of the material, and thereby the device’s efficiency and stability. Therefore, careful control of the crystallization process is key to achieving the optimum optoelectronic properties of the perovskite films. In solution-based film processing (Figure 2a), the use of the solvent dimethyl sulfoxide (DMSO) was a critical milestone in decelerating the crystallization of Pb and Sn perovskites through the formation of intermediate phases involving the metal halides and the sulfoxide.^94−96^ For the case of Sn perovskites, however, owing to their inherent propensity for faster nucleation, the effect of DMSO is insufficient to effectively retard the crystallization process.^98,99^ Indeed, Sn-based precursors strongly accelerate the crystallization process in mixed Sn−Pb perovskite films.^102^ Due to this rapid crystallization, mixed Sn−Pb perovskite thin films typically suffer from poor morphology with less-oriented grains, which in turn dramatically affects their optoelectronic properties. In addition, the different crystallization dynamics between Pb and Sn perovskites create competition between these two materials, inevitably leading to inhomogeneities in the distribution of the two metals throughout the film.^103^ This nonstoichiometric nature of the material is a source of trap states from tin interstitials in tin-rich spots and tin vacancies in tin-poor spots^104^ and leads to energy disorder inside the perovskite film.

Controlling the crystallization of the films is relatively less studied for the mixed Sn−Pb perovskite film,^105,106^ and the detailed mechanism is thus still largely being covered. Several attempts have been made using accustomed processing techniques, such as vacuum-assisted growth,^107^ gas-quenching,^108^ or two-step processing,^109^ to manipulate the crystallization kinetics. Other, more established protocols propose the use of 2D materials for controlling the growth and orientation of the grains,^110−112^ with close reliance on the perovskite compositions in some cases,^113^ and the addition of SnF_2_ to deliver more homogeneous nucleation of neat Sn-based perovskite films through the action of the F^−^ anion.^114^ In this aspect, reports on the use of chloride-containing additives suggest that this halide would have a similar role to the F^−^ anion.^115^ Additives containing pseudohalides,^116^ such as ammonium thiocyanate (NH_4_SCN)^117,118^ or lead(II) thiocyanate (Pb(SCN)_2_),^119,120^ have been applied to adjust the crystallization kinetics of the perovskite films, especially in combination with low-dimensional species,^121^ and/or the binding ligands.^122^ Despite the benefits of these strategies to control the crystallization of mixed Sn−Pb perovskites, the community still struggles to fabricate thin films with sufficient quality, considering the lower defect tolerance of these mixed perovskites compared to their conventional neat Pb counterparts. Thus, further efforts should be directed toward understanding the crystallization process of mixed Sn−Pb perovskites, in order to enable films with reduced grain boundaries, enhanced crystallinity with fewer intragrain defects, and a homogeneous distribution of tin- and lead-based units.

Perovskite inks are not a fully dissolved mixture of ions but are rather a colloidal dispersion by nature and contain complexes.^123^ This makes their crystallization a particularly complicated process, where the combination of classical and nonclassical nucleation theories introduces a number of possible nucleation and crystal growth pathways that cannot be straightforwardly ascribed to each specific perovskite case.^124−126^ Therefore, it is particularly relevant to gain a further understanding of the precursor colloidal dispersions and precursor chemistry during the early stages of perovskite crystallization. The nature of these colloids is going to determine the nucleation and crystal growth pathways of the perovskite material, and therefore it is critical to investigate their physicochemical characteristics and how they can be manipulated to control the crystallization process (Figure 2b, c). For this purpose, a series of traditional and novel techniques have been proposed in the literature. Conventional methods such as dynamic light scattering (DLS)^127^ and UV−vis spectroscopy^128^ can reveal important information on colloidal properties. However, these determinations are carried out indirectly, which could mislead researchers and result in inaccurate interpretations.^129^ Instead, small-angle X-ray (SAXS) and neutron scattering (SANS) are nondestructive techniques that provide critical structural properties of the perovskite dispersions, such as the size, shape, and structure of the colloids, while avoiding the limitations of DLS and UV−vis spectroscopy.^26,114,129−131^ In addition, small-angle scattering can be combined with other characterization methods to reveal further insights into the arrangement and interactions of metal halide species and additives in the precrystallization stages. For instance, ^207^Pb-NMR (nuclear magnetic resonance) can describe the chemical environment of the iodidoplumbates in solution to support the findings by SAXS.^26^ Although the use of NMR for studying perovskite colloidal dispersions is very scarce, we anticipate a high potential and versatility for such a simple, fast, and nondestructive technique. Other techniques also have the ability to uncover solution properties. For instance, cryogenic transmission electron microscopy (cryo-TEM) has found colloids in neat Pb precursor solutions to be crystalline, nonperovskite materials rather than amorphous materials.^132^ Identifying how colloidal properties affect precursor material evolution into perovskite material during the crystallization process would allow for control over the optoelectronic properties of the resulting thin films, describing the origin of defects and suppressing their formation from the solution stage.

The different polyiodide plumbates (PbX_m_^2−m^) and stannates (SnX_m_^2−m^) formed in the solution offer relevant information on the nature of these perovskite precursor solutions. The ligands present in the mixtures, i.e., solvent molecules, halides, and potential additives, compete to coordinate the metallic centers. Thus, their relative binding strength will define the valency and structure of these MX_m_^2−m^ species, which will consequently show as isolated complexes or even form colloids.^28,133^ Characterization of these solutions by absorption spectroscopy has revealed the ability of stronger binding solvents (e.g., DMSO > DMF) and halides (Cl^−^ > Br^−^ > I^−^) to decrease the number of high valency polyhalide metalates (i.e., lower m), providing the guidelines to control the properties of the precursor solutions. New solvent and halide mixtures have been proposed for successful crystallization tuning of neat Sn^79,134−137^ or even mixed Sn−Pb perovskites.^138^ Therefore, we would like to emphasize the great potential of structural modifications and solvent engineering approaches to address the current flaws in Sn-containing perovskite crystallization, avoiding the more common overdependence on additives. Unfortunately, most of the reports on absorption spectroscopy focus on neat Pb solutions, while examples for Sn-containing solutions remain scarce.^135,138−140^ In fact, the relevance of polyhalide metalate complexes in mixed Sn−Pb perovskite precursor solutions and how to manipulate them is often disregarded and, as a consequence, key aspects of these solutions remain unexplored. Thus, we predict exhaustive absorption measurement studies on Sn-containing perovskite solutions to be critical for the advancement of the processing of these materials.

One main challenge ascribed to Sn-containing perovskites processing is their faster crystallization kinetics with respect to Pb perovskites, as mentioned above.^6^ Particularly, Sn perovskites suffer from heterogeneous nucleation and rapid crystal growth. Most of the relevant studies reported in the field highlight the importance of increasing the stability and homogeneity of the generated nuclei, as well as adjusting their formation rate to crystal growth.^136,138,141−143^ Numerous strategies have, in fact, successfully controlled crystallization to some extent and improved thin film quality, but the fundamental reasons determining this nature inherent to Sn perovskites remain largely underexplored. The stronger Lewis acidity and different electronic structure of Sn(II) in comparison to Pb(II) are critically influencing the energetics and chemical characteristics (e.g., coordination, size, geometry, etc.) of the species that will be formed in solution and into a solid thin film.^144^ Studying them would not only be crucial for the development of neat Sn-based perovskites but would also offer highly relevant information about chemical properties, degradation processes, and thin film processing in mixed Sn−Pb perovskites. As we observed, the limitations encountered in the processing of high-quality mixed Sn−Pb perovskite thin films may stem from the fact that both their precursor solution chemistry and film crystallization dynamics are potentially dominated by Sn-based species. Here, we can selectively regulate the chemical environment of specific metal-based precursors in solution to control the different crystallization kinetics between the Sn and Pb perovskites. In this regard, additives like sulfate anion, with the ability to selectively coordinate Sn-based precursors, can slow the kinetics of the perovskite formation, adjusting it to become comparable to the Pb-based precursors.^145^ A balanced crystallization of both species leads to a more homogeneous distribution of the Sn and Pb species and consequently a higher uniformity in the energy distribution in the perovskite film. Based on the solubility difference of the Sn- and Pb-based perovskite species, in contrast, the vertical compositional gradient structure of the mixed Sn−Pb films has also been intentionally introduced by controlling the temperature of the antisolvent applied during the spin coating process.^146^ This gradient structure is supposed to provide a better energetic alignment between the perovskite and the charge transport layers. However, the thermal stability, e.g., under a temperature of 85 °C, of this structure may be an issue considering the ion movement of the metal cations inside the 3D mixed Sn−Pb perovskite films. We accordingly prospect that a rational design of this gradient structure could be realized by introducing 2D spacer(s) into the system to improve the thermal durability of the perovskite material, to some extent, while a reshape of the band structure, e.g., bandgap, of the material could be expected.^147^ Nevertheless, a deeper fundamental understanding is required in order to increase the number of available strategies to tackle/utilize the imbalance between Sn and Pb perovskite crystallization.

Moreover, up to now, there are few reports on intermediate crystallization for neat Sn or mixed Sn−Pb perovskites, and opposite to PbI_2_ in neat Pb materials, no much solvated crystal structures containing organic species have been reported yet, except for some cases of bidentate ligands like maltol.^12^ Some interesting species reported that may exist as intermediates are DMSO- and DMF-based SnX_2_ solvates: SnI_2_·DMSO,^141^ SnI_2_·2DMSO,^148^ SnI_2_·3DMSO,^6^ SnI_2_·DMF, 3(SnI_2_)·2DMF, SnBr_2_·DMF, SnBr_2_·2DMSO, SnCl_2_·DMF, and 2(SnF_2_)·2DMSO.^148^ The interactions established between the Sn 5s antibonding orbital and the donor orbitals,^149,150^ as well as the shorter Sn−O bond compared to Pb−O (for MX_2_-solvent) owing to the stronger binding by DMSO to Sn, lead to geometry differences between PbX_2_ and SnX_2_ solvates.^151−153^ The actual implications of this variation in the characteristics of the intermediates and the difficulty to identify them are yet to be defined, however. It is worth noting that intermediate phases have been found for mixed Sn−Pb perovskites, though they could not be unambiguously assigned yet.^138,154,155^ From here, some advanced techniques (Figure 2d), such as in situ grazing incidence wide-angle X-ray scattering (GIWAXS),^156^ in situ photoluminescence (PL),^157^ and adsorption spectroscopy,^158^ as well as liquid-phase TEM^159^ with the combination of matured single-crystalline XRD, may be key to shed light on the intermediate crystallization pathway of Sn-containing perovskites and bridge the gap between liquid precursors, crystallization, and final solid state of the films.^110^ However, one main obstacle to the applicability of these techniques to mixed Sn−Pb perovskites, particularly in the case of in situ GIWAXS, is the instability of Sn-containing materials to atmospheric agents. In this sense, adapting these techniques to the inert sample environments required by oxidizable species would open the door to new types of characterization and insights into these materials.

Nevertheless, current solution-based processing protocols, inherited from the processing of neat Pb perovskites, remain challenging to adapt to the Sn-containing materials.^160,161^ The explorations on epitaxial growth of perovskites may be proving this point.^81,162^ In these works, the perovskites with different compositions, including mixed Sn−Pb perovskites, lead to excellent quality thin films with a reduction of structural defects. The resulting films experienced a significant improvement in carrier mobility and recombination dynamics for their application in photovoltaic devices. Although the techniques presented in these works may not be readily transferable to general use, they highlight the actual potential of these materials and the importance of understanding and controlling the perovskite crystallization process. We also anticipate the strong potential of additive engineering strategies involving molecules with specific functional groups, for modulating mixed Sn−Pb perovskite colloids in the solution stage and positively influencing the crystallization process.^9^

### 2.2 Oxidation

Stability to ambient factors is critical for the industrial application of PSCs. While water, for instance, is a widely reported degradation source for metal halide perovskites,^163−165^ the instability of mixed Sn−Pb halide perovskite materials and devices are governed by the action of oxidant species on Sn(II)-based materials,^166−169^ mainly by atmospheric oxygen, and it can be accelerated by moisture.^170^ This is due to the thermodynamically favorable oxidation process of Sn(II), where the acquirement of two electrons by Sn(IV) has a positive standard reduction potential as low as 0.15 V. The origin of this critical difference between Sn and Pb elements stems from the lanthanide contraction affecting Pb, which is in the same group as Sn but in a higher period number.^171^ The full 4f subshell electrons present in Pb have a low shielding ability on outer subshells, not being able to compensate for the increase in the atomic number by 14. Thus, the more charged nucleus exerts a stronger attraction on the outer 6s orbital, stabilizing +2-oxidation state. This is not the case for Sn, which lacks the 4f subshell and can easily lose both 5s and 5p orbital electrons. In addition, this also causes the valence band maximum (VBM) of Sn perovskites to be shallower than for Pb perovskites^172^ and destabilizes the Sn−I antibond, contributing to the facile oxidation of Sn(II) into Sn(IV) and the formation of Sn(II) vacancies. According to the Frost−Ebsworth diagrams,^173^ we can observe that the oxidation of Sn(II) species is much more thermodynamically favorable than that of Pb(II) species. Moreover, the application of light or voltage can easily generate I_2_ from I^−^, which would have the ability to oxidize Sn(II). Severely harmful for neat Sn perovskites, this degradation is suppressed when blended with Pb in mixed Sn−Pb perovskite materials and devices, raising the oxidation reaction activation energy and slowing down the kinetics of the process.^104,174^ The oxidation mechanism necessarily involves several adjacent Sn(II) centers.^174^ Therefore, the intercalation of Pb centers in mixed Sn−Pb perovskites raises significant obstacles to the oxidation process. Further studies of the degradation pathways found that air exposure results predominantly in the formation of deep trap states, rather than electronic doping generally observed in neat Sn perovskites.^104^ This finding is in opposition to previously reported theoretical calculations, suggesting bandgaps free of deep trap states for mixed Sn−Pb perovskites.^175^ Previous findings by X-ray photoelectron spectroscopy (XPS) propose the formation of SnO_2_ and the consequent generation of defects, i.e., tin and iodide vacancies and tin interstitials, as the origin of these deep trap states.^176^ As a consequence, the monomolecular recombination of free carriers is accelerated, resulting in the decline of the optoelectronic properties of perovskite films. Thus, the presence of SnO_2_ will negetively impact the device performance, even at low concentrations, due to its high number of defect states.^177^ Concerning this, current analysis techniques of the Sn(IV) content (i.e., mainly XPS) do not have the sensitivity to detect the very low maximum concentrations of oxidized species that are tolerated for optimum cell performance (∼10^−8^ M),^79^ and thus seeking suitable analysis methods for the appropriate content range would aid the community to reach precise control on the materials.

**Figure 3:**
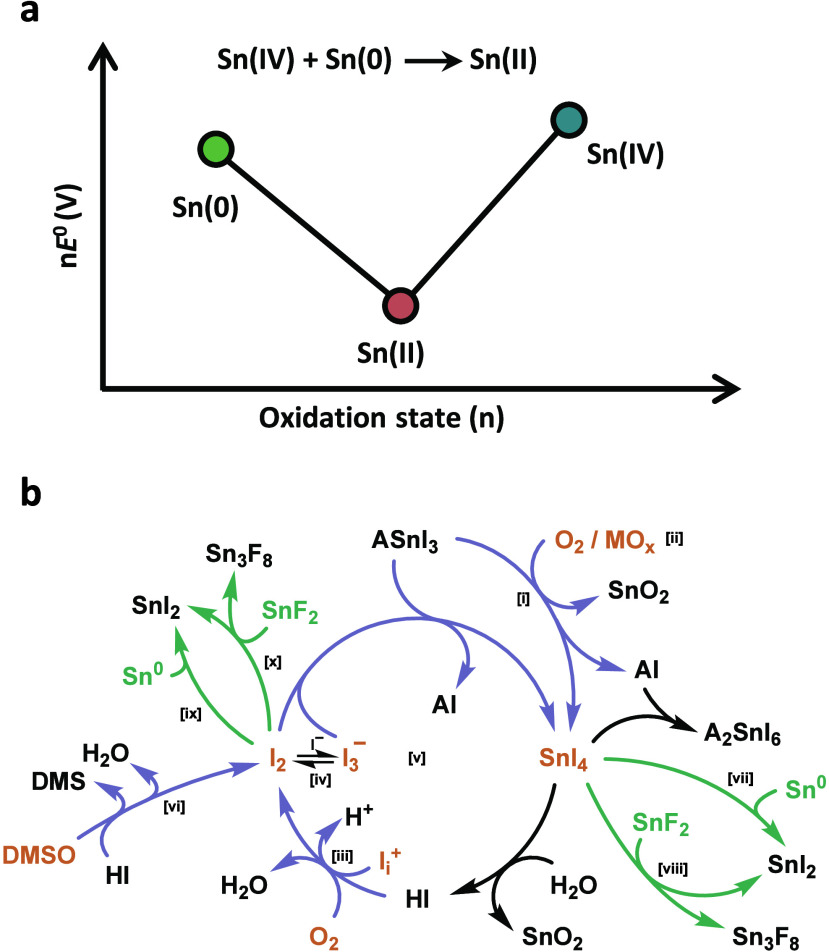
Oxidation. (a) Frost−Ebsworth diagram for tin demonstrating the favorable comproportionation reaction between Sn(IV) and Sn(0) to form Sn(II). Reproduced with permission from ref (144). Copyright 2023 American Chemical Society under CC-BY-NC-ND 4.0. (b) Reactions reported so far involved in the oxidation process of Sn materials, based on the cyclic degradation mechanism previously proposed.^178^ Molecules in orange represent oxidant species, while arrows in gray and green describe, respectively, oxidation processes and oxidant-removal processes through additives. (i) Oxidation of 3D Sn-based perovskite material ASnI_3_ by molecular oxygen, generating oxidized Sn-materials SnI_4_ or A_2_SnI_6_.^174,178,179^ (ii) Oxidation of Sn-based perovskite material by metal oxides as HTM, generating SnO_2_ at the interface.^180^ (iii) Oxidation of iodide by undercoordinated iodide ions (e.g., interstitial iodides, I_i_^+^) or molecular oxygen to the oxidant species iodine (I_2_).^179,181^ (iv) Triiodide formation reaction from iodine.^181^ (v) Cyclic oxidation mechanism of Sn-based perovskite material through the regeneration of SnI_4_ and I_2_ species under ambient conditions.^178^ (vi) Oxidation of iodide ions to iodine by DMSO.^182,183^ (vii) Comproportionation reaction of SnI_4_ and Sn^0^ species to SnI_2_.^184−186^ (viii) Selective complexation of Sn(IV) material by fluoride anions in SnF_2_, resulting in Sn_3_F_8_ mixed valence phase and regeneration of SnI_2_.^137,186,187^ (ix) Formation of SnI_2_ by the redox reaction between Sn^0^ and I_2_.^141^ (x) Reaction between I_2_ oxidant and SnF_2_ reductant to form SnI_2_ and Sn_3_F_8_ mixed valence phase.^187^

A simple and direct method to extend the stability of Sn-containing perovskite devices to extrinsic elements is to employ encapsulation technologies. These strategies significantly alleviate the instability of these materials by preventing oxygen from entering the device. While current industry standard encapsulation methods are about sufficient for Pb-based devices,^188^ their suitability for Sn-containing devices still needs to be further studied. These materials will certainly require more strict conditions since even small quantities of oxygen absorbed on the perovskite surfaces can already cause the formation of detrimental species.^176^ Therefore, encapsulation may provide just a partial solution to the oxidation issue. To address this, the community should develop device fabrication protocols that inherently increase the stability of the material to oxidation, in parallel with the combined implementation of adapted encapsulation strategies. Here, the simultaneous optimization of the different surfaces in the Sn-containing materials can make a critical difference in tackling their unstable nature. As mentioned before, perovskite surfaces in the film (grain boundaries, interfaces) are a major site of degradation and efficiency loss. Current strategies propose the use of various additives,^189^ such as SnF_2_,^190,191^ Sn(0),^184,186^ and Pb(0)^192^ species, some reductants/antioxidants,^86,146,193−195^ V^3+^/V^2+^ ionic pair as a redox shuttle,^196^ and the electron-withdrawing ligand that improves the redox potential of the tin adduct.^197^ They effectively reduce the content of Sn(IV) in the precursor solution and thus suppress the *p*-doping in the films caused by the forming of the Sn(II) vacancies. For example, the SnF_2_ addition in neat Sn and mixed Sn−Pb perovskite films makes a significant reduction in the background hole density due to the reduced Sn(II) vacancies.^198^ Consequently, the carrier lifetimes are elongated, the energetic disorder is decreased, and Burstein−Moss shifts^199,200^ are reduced for the films. In addition, fluoride anions in SnF_2_ selectively capture Sn(IV) species and eliminate them from the bulk,^114^ which was later confirmed by DFT calculations, suggesting the sequestration of Sn(IV) through the thermodynamically favorable formation of mixed valence Sn_3_F_8_.^187^ Doping with heterovalent metallic cations, like Ag^+^ and Ga^3+^,^155,201^ has also opened another interesting way to increase the antioxidative character of the mixed Sn−Pb perovskite material. Unfortunately, the currently established additive-based strategies fall short of fully eliminating these trap states, highlighting the need for advanced strategies that more strongly inhibit the oxidation-related defects simultaneously in grain boundaries and interfaces of mixed Sn−Pb perovskite films.

So far, our discussion has touched upon the atmospheric oxygen-driven decomposition, which is the main contributor to the oxidation of Sn(II) species.^176^ However, iodine species (I_2_ and triiodide ion, I_3_^−^) are other oxidants and an important source of defects to be considered because they have been reported to be formed as a decomposition product of iodide-containing perovskite materials and under the presence of illumination and bias (applied voltage).^176^ It also takes part in the cyclic degradation of tin halide perovskites, due to the formation and regeneration of SnI_4_.^178^ In addition, the conventional solvent to process these materials, DMSO, can also be reduced in the presence of iodide ions, generating in turn iodine-based oxidant species that can degrade Sn(II) perovskite material.^78,79,183^ The extent of this oxidation and its actual impact on the device performance is still to be determined and might be particularly critical for neat tin perovskites due to their higher ratio of tin content. Promising works in neat Sn PSCs point out the potential benefits of DMSO-free fabrication processes.^134,136,168,202^ A recent study proved its efficacy for mixed Sn−Pb perovskites, where the utilization of a DMPU (*N,N′*-dimethylpropyleneurea)-based solvent system largely reduced the formation of Sn(IV).^138^ Finally, the charge transport materials are an additional source of oxidation of the perovskite component. Metal oxide semiconductors, such as NiO_X_ and TiO_2_, have active species that can induce Sn(II) oxidation at the perovskite interface.^180,203−205^ In Figure 3, we summarize every reaction reported so far related to the oxidation process of Sn materials. We listed the different sources of oxidant species O_2_ and I_2_ and coupled them with the cyclic oxidation mechanism of ASnX_3_ materials.^178^ Moreover, we have also included the main antioxidant strategies and how they influence the degradation process by removing oxidized species from the cycle.

Oxidation of the mixed Sn−Pb perovskite material is a complex process that involves a considerable number of sources, such as oxygen from the air, metal oxide contacts, or I_2_ from the perovskite itself or the influence of DMSO solvent. In addition, each of them would require a specific strategy to be dealt with. The atmospheric oxygen may pose the biggest challenge, due to the difficulty of fully avoiding its insertion in the material. While the combination of current additive-based antioxidants with proper encapsulation techniques could lead to encouraging results, there exists the risk they may dissatisfy the high standards required to keep Sn(II) stable.^168^ Therefore, future efforts should be aimed at increasing the intrinsic stability of Sn-containing materials through structural and process modifications. Moreover, the strongest impact of oxidation happens at the perovskite interfaces, due to their higher exposure and their more detrimental effect on device performance. Thus, the processes to be designed by the community should be directed not only to inhibiting the oxidation process but also particularly to safeguarding these sites in solar cell devices.

### 2.3 Mass Loss

To analyze the viability of PSCs for practical use, the evaluation of perovskite degradation upon external stress, induced by factors such as temperature, light, humidity, electrical bias, and radiation, is essential. For neat Pb PSCs, the material loss has been widely investigated and is now rather well understood, as well as for neat Sn PSCs, where material loss is largely linked to oxidation.^206^ However, mass loss in mixed Sn−Pb perovskites is more complex due to the more convoluted material chemistry. Still, several attempts have been made to investigate this critical aspect.

**Figure 4:**
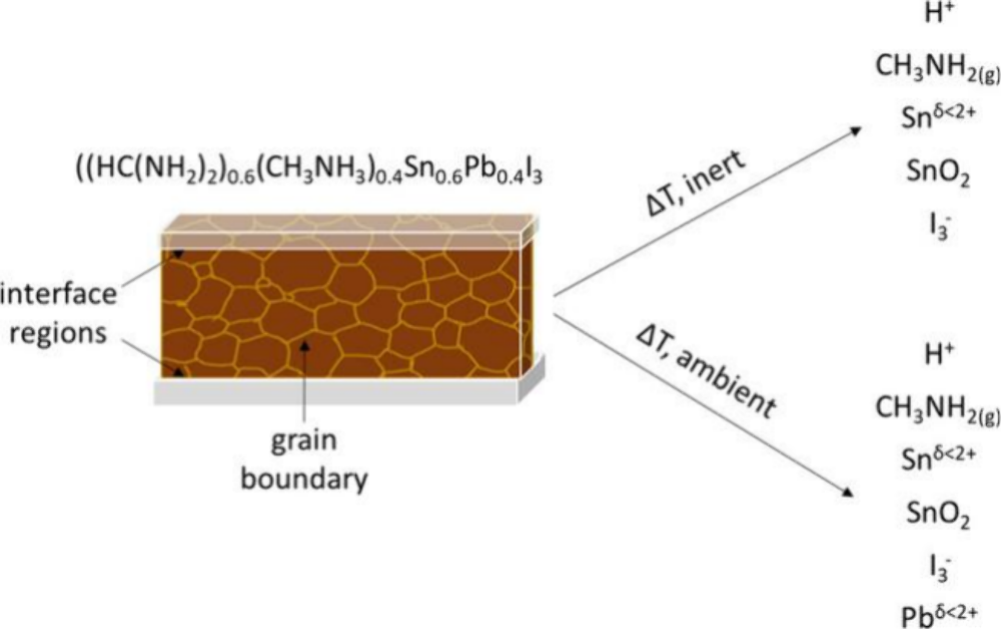
Mass loss. Schematic of proposed chemical degradation mechanism in perovskite surface regions, including interfacial regions between the perovskite active layer and charge collective layers as well as grain boundaries. Reproduced with permission from ref (179). Copyright 2020 American Chemical Society.

Mass loss in mixed Sn−Pb PSCs is tightly linked to redox chemistry,^174^ showing the dependence of mass loss mechanisms on the Sn/Pb ratio, with the activation energy in Sn-rich perovskites (>50% Sn) being lower than that for perovskites where Sn sites are surrounded by a larger number of Pb sites. Moreover, surfaces are key in this degradation mechanism and, in this sense, the removal or modification of the conventional hole transport contact, poly(3,4-ethylenedioxythiophene) polystyrenesulfonate (PEDOT:PSS), can lead to more stable cells, which are less prone to oxidation and therefore suffer less mass loss.^207^

To identify structural changes within mixed Sn−Pb perovskite devices under operation, synchrotron-based operando XRD can probe changes in the crystal structure of the perovskite absorber.^176^ Interestingly, the combined stressors of heat, light, and electrical bias induced no change in the bulk crystal structure. The film surface, on the other hand, degraded increasingly fast due to activated corrosion processes, confirming that detrimental chemical reactions dominate degradation in mixed Sn−Pb PSCs. Organic cations are lost at the surface through deprotonation, a process that is accelerated at elevated temperatures (Figure 4). XPS analysis showed the existence of I_3_^−^ species, suggesting that photochemical reactions of I^−^ with photoactivated/generated holes are also likely to occur, which eventually culminates in the loss of I_2_ at the surface. Finally, regarding the loss of the metal cations from the lattice, this commonly originates from the oxidation of Sn(II) to SnO_2_, while Pb(II) can reduce to Pb(0) under ambient-catalyzed conditions. While the combined presence of oxygen and humidity accelerates the oxidation process of Sn(II),^170^ the intentional hydrolysis of the superficial Sn(IV) with H_2_O can, however, form a thin SnO_X_ layer, which decreases Sn(IV) defects and forms a passivated and n-type surface that contacts desirably with the electron transport layer (ETL).^208^

Temperature is a critical parameter affecting the degradation mechanisms. Most degradation processes, be them triggered by light, charge density, electric field, or oxygen, are chemical reactions that are thermally activated, with increasing temperature accelerating the degradation rates. For instance, the oxidation process of Sn(II) by DMSO is too slow to be detected at room temperature but becomes very evident under elevated temperatures, e.g., over 100 °C.^183^ Meanwhile, the traditional A-site cation methylammonium (MA^+^) has low thermal stability^209^ and is known to leave the film as various degradation products, including methylamine, ammonia, and hydroiodic acid.^210^ These gaseous materials will leave the perovskite film and react with the other materials in the device structure, such as the metal electrode. Thus, removing MA^+^ from the perovskite material, neat Pb perovskite included, increases the thermal and long-term operational stability of the devices.^207,209,211^

Besides stressors such as temperature, electrical bias, and light, the impact of proton irradiation on mixed Sn−Pb PSCs, an aspect that is specifically important for their prospective implementation in space, has also been investigated. Interestingly, mixed Sn−Pb PSCs are remarkably radiation tolerant^212,213^ and far outperform other thin film technologies in this respect, making them ideal candidates for space applications.

Overall, the chemical degradation in mixed Sn−Pb PSCs is surface-dominated, and the oxidation of Sn(II) in the perovskite is a process that occurs in combination with the oxidation of I^−^ and the reduction of metals. The initiation of these redox reactions depends on local electrochemical potentials, which are in turn defined by a complex combination of defects, the presence and concentration of mobile species, and additional decomposition products. Thus, it is essential to develop strategies to increase the quality of the surface (decreasing the superficial defects) and protect it from further degradation, such as surface engineering with post-treatments or the insertion of passivating thin layers. Furthermore, parallel degradation mechanisms involving the organic A-site component will also influence the mass loss and defect generation in mixed Sn−Pb PSCs. With MA^+^ being the main component affected by thermal decomposition, the development of inorganic and MA-free mixed Sn−Pb PSCs is necessary to achieve long-term stable devices that could meet the criteria for practical applications.

### 2.4 Ion Movements and Charge Carrier Transport

**Figure 5:**
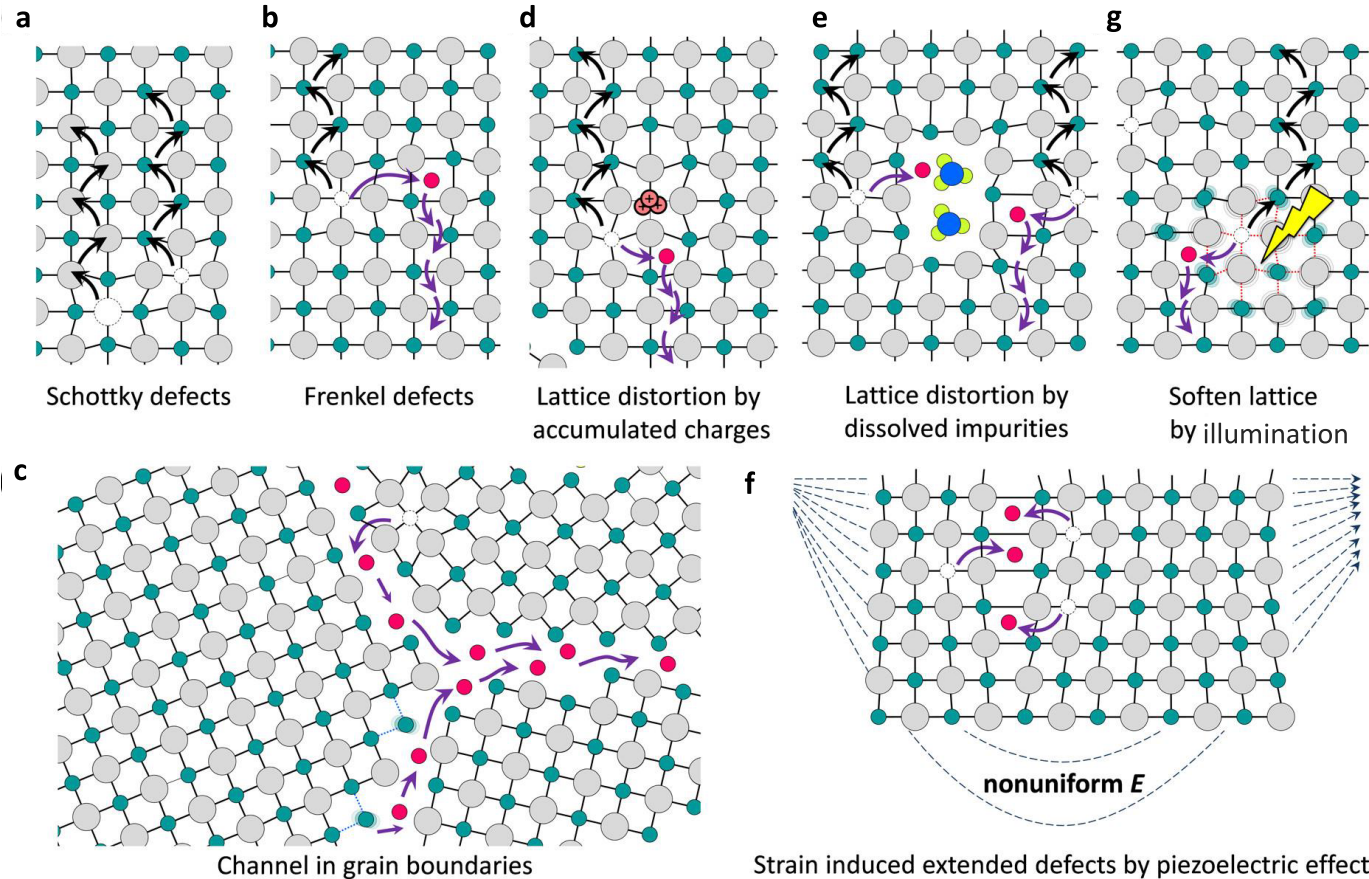
Ion movement. Illustration of the ion migration pathways enabled by (a) Schottky defects (b) Frenkel defects, (c) open space and (d−f) wrong bonds at grain boundaries. Lattice distortions due to (d) accumulated charges, (e) dissolved impurities, (f) nonuniform strain caused by the piezoelectric effect, and (g) softened lattice caused by the light illumination induced bond weakening. Reproduced with permission from ref (7). Copyright 2016 American Chemical Society.

The presence of mobile ions in metal halide perovskites of different compositions has been well established and linked to hysteresis and device (in)stability.^214−220^ Although there is still debate about exactly which ions are moving and how high the corresponding mobile ion densities are,^221−223^ the important role that mobile ions play in PSCs is generally well recognized (Figure 5). In addition to causing phase instabilities and chemical decomposition of the perovskite, mobile ions can influence the performance of PSCs by altering their electronic properties. For example, mobile ions can cause light-soaking effects, leading to a change in open-circuit voltage (*V*_OC_) over time upon exposure to light.^224^ Furthermore, they can drastically impact the device short-circuit current density (*J*_SC_) and fill factor (FF).

Recently, a combination of transient charge extraction and photoluminescence measurements demonstrated that both neat Pb and mixed Sn−Pb PSCs suffer from current losses during the first seconds of operation, caused by the movement of mobile ions in the devices and subsequent field screening.^220,225^ Thiesbrummel and Le Corre et al. showed that these current losses were a consequence of band flattening caused by the redistribution of mobile ions and the consequent screening of the internal electric field. However, ion-induced shifts to the energetics at the charge extraction contacts were not considered, which may also play a role in the reduction in charge extraction efficiency. In mixed Sn−Pb PSCs, besides the mobile ions which are also present in neat Pb PSCs, there might be additional ions involved due to the oxidation of perovskite. Mobile ions such as FA^+^ or I^−^ are likely formed upon oxidation of Sn-containing perovskites, which would mean that oxidation of Sn(II) to Sn(IV) in the perovskite could increase mobile ion densities, leading to increased current losses.^174^ Furthermore, the reduced absorption coefficient of mixed Sn−Pb perovskites compared to neat Pb ones,^225^ as a consequence of the lower exciton binding energy in the Sn-containing perovskites, means that thicker perovskite layers need to be used, further enhancing the effects of mobile ion-induced band flattening on the output current density. On the other hand, however, recent publications do suggest that incorporating Sn in perovskites reduces the initial impact of mobile ions on device performance by increasing activation energies that ions would need to overcome to move, thereby reducing the diffusion rate of mobile ions.^226,227^ Nevertheless, the extent to which ion movement is present in Sn-containing perovskites, as well as the mobility of these ions, may depend strongly on their composition and thin film quality, which could be easily influenced by the use of additives, such as RbI.^228^ Finally, there are indications that mobile ion densities in Sn-containing perovskites strongly increase upon aging, similarly to those in their neat Pb-based counterparts, as well as upon oxidation.^229^ All in all, we anticipate additional research efforts in this direction, which will eventually reveal the critical aspects of ion migration in mixed Sn−Pb perovskites.

Mobile ion density and the active layer thickness, however, are not the only factors determining the ion-induced current losses in PSCs. The quality of the interfaces plays a large role in determining the eventual ion-induced current losses. Improved interface quality can significantly reduce the impact of mobile ions on the device performance,^223^ for example, by inhibiting mobile ions from diffusing into the transport layers^230^ or reducing nonradiative recombination at the interfaces.^231^ Charge transportation and extraction can be limited by defect-induced trap states. These traps mainly accumulate at the surfaces, including both the grain boundaries and the perovskite interfaces with the charge extraction layers. The interfaces with the charge carrier selective contacts are also a source of losses. The energy level mismatch induced by the transporting layers is largely associated with the nonradiative recombination of the accumulated (minority and majority) carriers at the interfaces,^232^ stressing the importance of interface engineering to overcome this loss mechanism. Another way for interface improvement is using doped transport layers that can increase the internal voltage over the perovskite layer, significantly reducing interface recombination.^233^ Both the reduced interface recombination and the increased internal field reduce the detrimental effect of mobile ions. Generally, the more pronounced the nonradiative recombination is in the device (typically limited by the interface), the more the device will be impacted by ion-induced field screening and the corresponding reduction in charge carrier extraction efficiency.

Overall, ion movement phenomena are present in mixed Sn−Pb perovskites, with the additional contribution from Sn(II) oxidation, and are responsible for current losses. However, current reports indicate that intrinsic ion migration in freshly prepared perovskite films may be reduced with increasing Sn content. Furthermore, the interfaces largely influence the ion-induced current losses. We forecast that the mobile ions present in the perovskite might largely stem from grain boundaries and interfaces, where activation energies for the creation of mobile species might be lower than in the bulk. These results once again underline the importance of interface optimization in mixed Sn−Pb PSCs.

### 2.5 Section Summary

Perovskite surfaces play a critical role in the performance of optoelectronic devices. Defects in these surfaces, namely, grain boundaries and interfaces, are particularly detrimental to the device operation, leading to profound nonradiative recombination and harming charge transport and extraction. On the one hand, the crystallization process dictates the final quality of the perovskite thin film. Tin species have a natural tendency to crystallize faster and in a hard-to-control manner, which competes with Pb species in the crystallization of mixed Sn−Pb perovskites and leads to morphological flaws and unoriented grains, generating, in turn, a significant variety of defects. Thus, we propose further research in the understanding of the perovskite solution properties and its evolution into thin films to better control the crystallization process, with emphasis on the nature of colloids and the stabilization of intermediates. On the other hand, intrinsic material instability to oxidation, mass loss mechanisms, and ion movement phenomena in materials and devices are the other main origins of surface defects and consequent device performance decline. Particularly, the oxidation and mass loss at the surface region of the material could lead to the formation of extraction barriers that prohibit charge carrier extraction, leading to the loss of cell efficiency. In the same line, mobile ions in metal halide perovskites can lead to inefficient charge extraction at the interfaces. Overall, the above-mentioned sources of imperfections will ultimately have their most harmful consequences at the interfaces, with a concentration of defects in these sites. Such treatments will ultimately improve the stimuli resistance,^97^ reduce the photochemical reactions and suppress movements as well as reduce their impact on device performance, enabling all-perovskite tandem devices with excellent long-lasting performances that are suitable for commercialization.

## 3 Interface Engineering for Solar Cells

Generally, planar PSCs are assembled in either a p-i-n or n-i-p device structure.^234^ Like the conventional neat Pb PSCs, the first series of mixed Sn−Pb PSCs were made with the n-i-p architecture. In 2014, two groups independently fabricated n-i-p devices with PCEs (power conversion efficiencies) above 4 and 7%.^235,236^ Although several more attempts have been made since then,^81,232,237−240^ the best PCE for mixed Sn−Pb n-i-p PSCs is still just around 16%,^241^ far below the current record of close to 24% realized with p-i-n devices.^9,77,242^ This efficiency gap is caused mainly by the detrimental chemical reactions between the charge transport material and the perovskite component in n-i-p devices. For example, it has been shown that oxygen vacancies from metal oxide *n*-type semiconductors,^203−205^ such as SnO_2_ and TiO_2_, induce Sn(II) oxidation at the ETL/perovskite interface.^180,203−205^ Moreover, the degradation of the perovskite may also be triggered by hygroscopic dopants (i.e., Li or Co salts) in the conventional hole transport layer (HTL) used in n-i-p devices,^232,243^ 2,2′7,7′-tetrakis-(*N*,*N*-di-*p*-methoxyphenylamine)-9,9′-spirobifluorene (spiro-MeOTAD). As a result, the charge extraction at the perovskite interfaces with the charge transporting layers is largely hindered, leading to strongly limited device efficiencies. Generally, the passivation of the metal oxide layer, which has been frequently demonstrated with an organic *n*-type semiconductor, like PC_61_BM^240^ and C_60_-SAM,^239^ is crucial for the growth of high-quality mixed Sn−Pb perovskite films. Likewise, interface modification and new HTL implementation could be an efficient way to mitigate interface recombination and improve the performance of the n-i-p devices.

**Figure 6:**
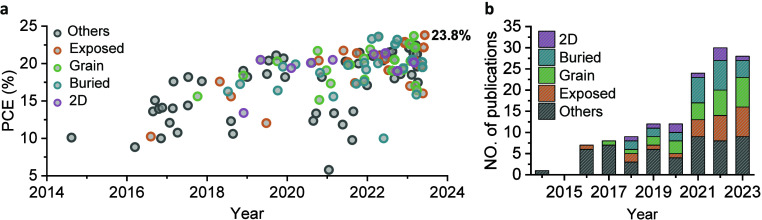
Interface engineering for solar cells. (a) Efficiency progress of mixed Sn−Pb PSCs with p-i-n structure, depending on the surface that was modified. The best-performing cell for each publication was included in the graph. (b) Stacked bar chart of the publications of mixed Sn−Pb PSCs classified with the interface-related treatment in each year. Exposed, grain, buried, and 2D denote exposed surface modifications, grain boundary modifications, buried surface modifications, and 2D capping strategies, respectively. The data were updated by June 10, 2023.

Because of their more widely implemented use—especially for all-perovskite tandems—and higher efficiencies as well as better device stability, in this section, we mainly focus on the devices fabricated with the p-i-n architecture, which has several advantages for the manufacturing of large-area modules^244^ and flexible electronics.^245^ As for the n-i-p architecture, the first p-i-n mixed Sn−Pb PSC was also reported in 2014, with a PCE of around 10% with the perovskite composition of MAPb_0.85_Sn_0.15_X_3_.^115^ Since then, significant efforts have been made regarding the optimization of the 3D perovskite composition and stoichiometry,^31,246−260^ especially via tuning the ratio of Sn(II) and Pb(II) cations.^261−270^ In the current stage, half Sn(II) and half Pb(II) content for the B-site cation is the most common combination because of its good optoelectronic quality and desirable bandgap for photovoltaic applications, especially as the rear absorber for the all-perovskite tandems.^269^ Here, we summarize the results from recent surface modification reports based on the single-junction mixed Sn−Pb PSCs and various related all-perovskite tandems. Each section differentiates each type of surface in the perovskite films and the specific characteristics and treatment requirements. In particular, we consider the top exposed surface, the grain boundaries, the bottom buried surface, and surfaces capped with 2D phases (Figure 6). We summarize some representative works and main strategies employed for each surface and point out the directions to investigate in the future to further improve these interfaces.

### 3.1 Exposed Surface

The most vulnerable surface of the perovskite films would be the exposed surface, due to its relatively extended period of contact with the atmosphere during the cell fabrication. Meanwhile, the p-doping in Sn-containing perovskites favors the accumulation of Sn(IV) at the surface, which in turn acts as electron traps that promote recombination and lattice degradation toward secondary phases.^271^ Degradation at the exposed surface will also negatively affect the charge extraction at the top contact, i.e., the electron extraction of the ETL in the case of a p-i-n cell. In addition, C_60_ molecules commonly employed in the p-i-n cells act as deep trap states when in direct contact with the perovskite films.^272^ All these things considered, engineering this surface to enable efficient charge transport and reduce interface recombination is crucial to achieving high device performance.^273,274^ The interface could be successfully improved by (1) improving the quality of the perovskite films (smoothness and crystallinity), (2) reducing the Sn(IV) content at the surface, (3) passivating the defects at the surface, and (4) altering the surface electronic properties toward an electron-transport favorable character in p-i-n cells. Theoretically, polishing the surface with chemical^9,275^ or mechanical^276^ approaches or even using lasers^277^ would allow for the removal of the defective nanostructures at the exposed surface. In addition, molecules that ensure effective chemical interaction with the perovskite lattice and efficient carrier extraction would be suitable for perovskite post-treatment at contact with extraction layers.

#### 3.1.1 Protection from Oxidation

**Figure 7:**
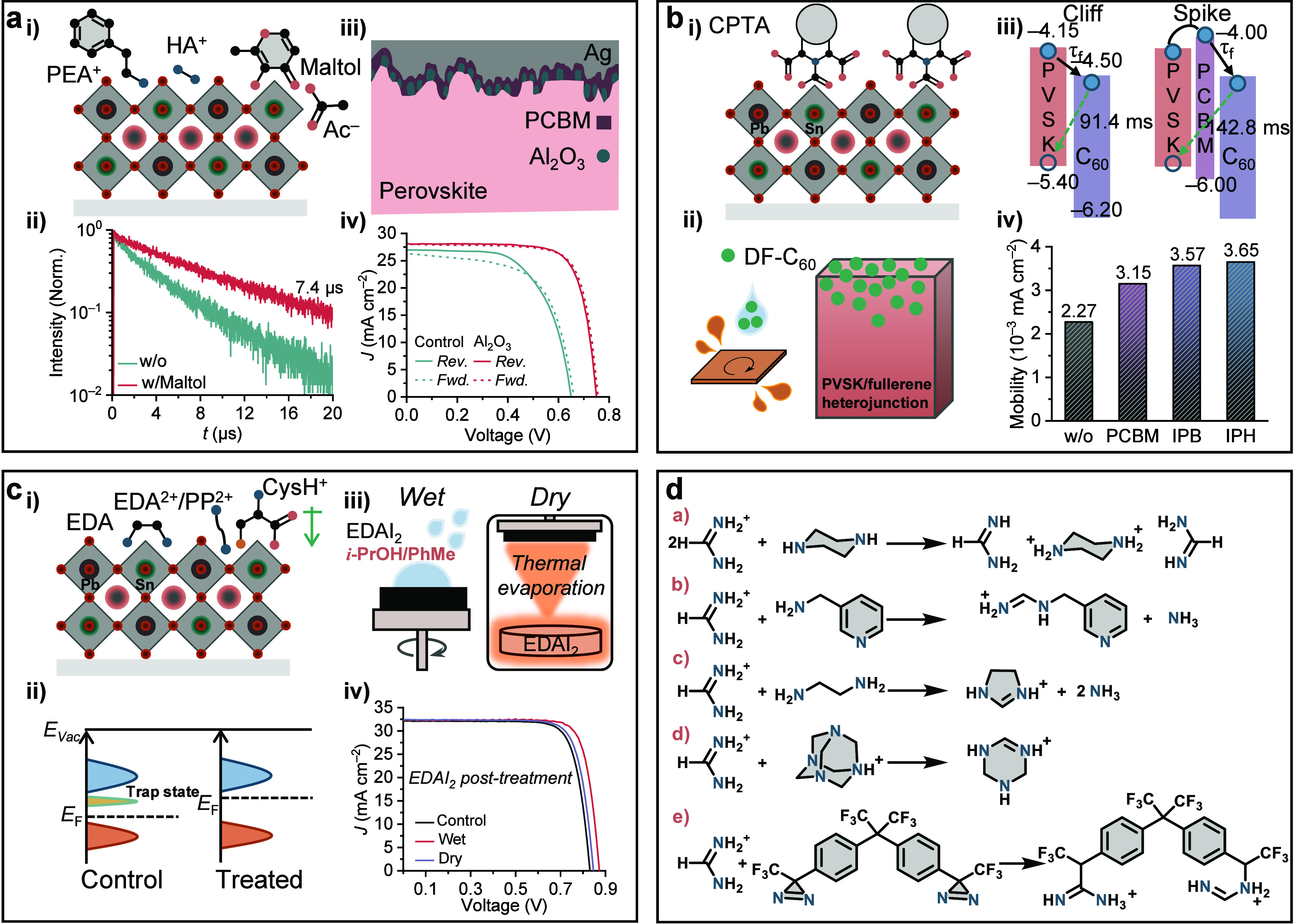
Exposed surfaces. (a) (i) Illustration of the mixed Sn−Pb perovskite films with different post-treatments. PEA^+^: phenethylammonium; HA^+^: hydrazinium; Ac^−^: acetate. (ii) Time-resolved photoluminescence (TRPL) decay curves of the perovskite films fabricated without and with maltol post-treatment on quartz substrates. Reproduced with permission from ref (12). Copyright 2021 Royal Society of Chemistry under a Creative Commons Attribution-NonCommercial 3.0 Unported License. (iii) Schematic illustration of how a PCBM layer deposits on a rough mixed Sn−Pb perovskite layer with Al_2_O_3_ nanoparticles post-treatment. (iv) *J*−*V* curves under simulated AM 1.5G illumination in forward (*Fwd.*) (*J*_SC_ to *V*_OC_) and reverse (*Rev.*) (*V*_OC_ to *J*_SC_) scan directions of the champion control and the champion device with an optimized Al_2_O_3_-nanoparticle interlayer thickness. Reproduced with permission from ref (30). Copyright 2023 Wiley-VCH under the terms of the Creative Commons Attribution License. (b) (i) Schematic illustrations of CPTA binding to the exposed Sn(II) at the surface of mixed Sn−Pb perovskite films. (ii) Graded heterojunction of mixed Sn−Pb perovskite films and DF-C_60_ fullerene introduced through the antisolvent. (iii) Schematic showing the charge extraction and recombination processes without and with the PCBM layer. τ_f_ shows the forward injection time from the mixed Sn−Pb perovskite films to C_60_. Reproduced with permission from ref (40). Copyright 2018 American Chemical Society. (iv) Comparison of electron mobility μ in electron-only devices with different interlayers. μ was determined by the space-charge-limited-current (SCLC) method. Reproduced with permission from ref (66). Copyright 2022 Wiley-VCH under the terms of the Creative Commons Attribution License. (c) (i) Illustration of the mixed Sn−Pb perovskite films with different post-treatments. EDA binds to the metal center of the perovskite. EDA^2+^, PP^2+^, and CysH^+^ create the desirable surface dipole that facilitates electron extraction. EDA: ethylenediamine; EDA^2+^: ethylenediammonium; PP^2+^: piperazine-1,4-diium; CysH^+^: cysteinium. (ii) Electronic structure of the mixed Sn−Pb perovskite films fabricated without and with the post-treatments. (iii) Universal EDAI_2_ post-treatment for p-i-n PSCs through both wet and dry processes. The mixed solvent of IPA (*i*-PrOH) and toluene (PhMe) is important for wet processing.^9^ (iv) *J*−*V* curves under simulated AM 1.5G illumination of the champion control and the champion device with optimized wet and dry EDAI_2_ post-treatments. Reproduced with permission from ref (72). Copyright 2022 American Chemical Society. (d) Representative chemical reactions disclosed in the perovskites for solar cell application. (a) Proton transfer reaction between FA^+^ and piperazine.^80^ (b) Condensation reaction scheme of FA^+^ and 3-APy.^61^ (c) Reaction of FA^+^ with EDA to produce Imn^+^ and ammonia.^50^ (d) HMTA reaction with FA^+^, leading to tetrahydrotriazinium (THTZ-H^+^).^82^ (e) Reaction between 3,3′-((perfluoropropane-2,2-diyl)bis(4,1-phenylene)) bis(3-(trifluoromethyl)-3*H*-diazirine) and FA^+^ generating the product with newly-formed covalent bonds.^83^

Post-treatments of perovskite crystallized films, or precrystallized films during the quenching step,^278^ are highly effective strategies for increasing the robustness of the material and its resistance to degradation from atmospheric O_2_, as well as removing oxidized species that existed at the surface. The newly-formed interfaces can block the invasion of air into the perovskite layer, and desired chemical bonds built during the process may also increase the intrinsic resistance of the material. The most commonly employed chemicals for post-treatment in the metal halide PSCs community are ammonium salt, e.g., phenethylammonium (PEA^+^)-based materials.^279,280^ Similar to its effects in neat Pb PSCs (Figure 7a-i),^279^ post-coated PEA^+^ can also improve the performance of mixed Sn−Pb PSCs.^281−283^ Due to the hydrophobicity and superior A-site binding capacity of PEA^+^, the modified films tend to be more robust with reduced defect states and decreased Sn(IV) concentrations at the surface. Besides A-site substitutes, strong X-site binding species, e.g., pseudohalide acetates (Ac^−^), are also effective in inhibiting the oxidation of Sn(II) and suppressing ionic migration, as their coordinating energy with Sn(II)/Pb(II) is larger than that for the I^−^ anion.^283^ However, ammonium ligands or pseudohalides that can form 2D phases require careful control as the 2D phase will probably introduce an energy level that is unfavorable for the carrier extraction at the interface in the p-i-n devices.^284^ Besides aiding in the passivation of the perovskite surface, some ammonium salts can also introduce a second crystal growth via Ostwald ripening, leading to a significant increase in grain size.^285^ For example, the grain size of the films exposed to methylammonium chloride (MACl) vapor can be increased to reach over 1 μm during the post-treatment, yielding polycrystalline films with improved quality.^106^ The PCE of the resultant cells was improved along with device resistance to air: Unencapsulated mixed Sn−Pb PSCs maintained their full performance after 150 h at 85 °C in air. There are also several different molecules that have the ability to reduce the amount of Sn(IV) or suppress its formation, such as hydrazinium (HA^+^),^286^ dopamine cation (DAH^+^),^287^ and borohydride-based materials,^288,289^ or maltol, a metal-chelating compound, which was used to produce perovskite films with carrier lifetimes of over 7 μs, which were later implemented to fabricate devices with PCEs over 21% (Figure 7a-ii).^12^

Developing chelating molecules that efficiently passivate and stabilize the fresh films, without inducing unfavorable carrier recombination, offers an effective way to improve the performance of mixed Sn−Pb perovskite electronics.^290^ On the other hand, electrical shunts might be another serious issue for an efficient device considering that mixed Sn−Pb films present a high degree of roughness, especially when a thin ETL is deposited in the following via spin coating (Figure 7a-iii, iv). In order to reduce the layer roughness and improve the conformality of subsequently coated ETLs, an extra layer might be required. Recent work shows a successful example of this, using an ultrathin noncontinuous Al_2_O_3_ nanoparticle layer to improve the efficiency and stability of the mixed Sn−Pb perovskite devices.^30^ By treating the buried or exposed surface of the perovskite films, researchers in the field have widely used Al_2_O_3_ and some other conventional metal oxides as thin interlayers, either with mesoporous or dense continuous form, for improving the stability and efficiency of PSCs.^63,291−295^ Regarding this particular successful application of Al_2_O_3_ nanoparticles in mixed Sn−Pb PSCs, the reason behind this would be the higher stability of Al_2_O_3_ compared to other metal oxides, such as TiO_2_ and NiO_X_, thanks to the stronger metal−O bond and less redox reactivity.^296−299^

**Figure 8:**
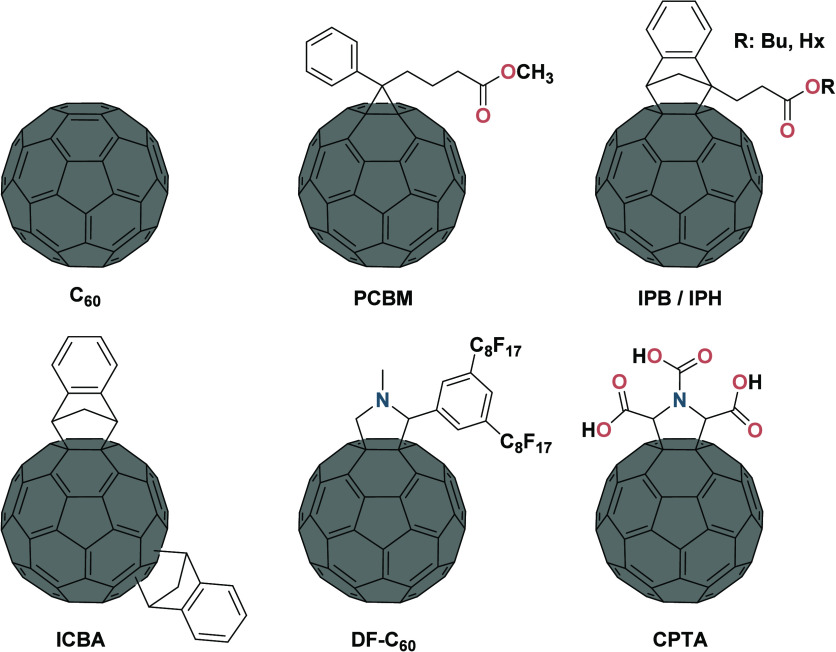
Chemical structures of C_60_ and its derivatives that have been employed in mixed Sn−Pb PSCs.

#### 3.1.2 Defect Passivation and Energy Structure

The facile oxidation of Sn(II) and the iodide ions lead to the generation of an abundance of defects at the perovskite surface, such as tin and iodide vacancies, as well as interstitials. Iodide vacancies generally exist in all kinds of I-containing perovskite films. In addition, halide anions are particularly mobile, especially at elevated temperatures. Theoretical calculations show that in mixed Sn−Pb perovskite polycrystalline films, iodide ions centered with Sn can be more easily detached compared to those centered with Pb,^80^ suggesting that the surfaces of tin-containing perovskites will present a higher density of exposed metal(II) centers compared to lead-containing perovskites. Apart from that, various site vacancies and interstitial states exist at the surface that also alter the surface energetic states of the films.

##### 3.1.2.1 Fullerene Derivatives

The vast amount of available knowledge on surface and defect chemistry in mixed Sn−Pb perovskites offers the potential to design passivation strategies that target specific surface defects through functionalized molecules. Fullerenes are versatile electron-transport molecules, typically applied on top of perovskite films,^300,301^ that can bear a variety of functional groups for specific purposes. Their flexibility and adaptability grants them enormous potential for improving the perovskite top interface. An example of a fullerene that is used for interface improvement in mixed Sn−Pb perovskites is the carboxylic group-containing fullerene derivative C_60_ pyrrolidine tris-acid (CPTA) (Figure 7b-i).^80^ XPS characterization showed that CPTA predominantly binds to Sn sites rather than to Pb sites, owing to the predominant exposure of the Sn sites at the film surface. The n-type nature of the fullerene derivatives also provides the treated films with superior electron extraction at the top surface. Consequently, PCE values of up to 22.7% were achieved for the devices fabricated using CPTA treatment, with *V*_OC_ values approaching 0.90 V, reaching a minimum voltage loss of ∼92% of the radiative limit for a ∼1.26 eV bandgap. Previously, fluoroalkyl-substituted fullerene *N*-methyl-2-(3,5-bis(perfluorooctyl)phenyl)-3,4-fulleropyrrolidine (DF-C_60_), in combination with indene-C_60_ bis-adduct (ICBA) as ETL, presented excellent passivation effects and enhanced surface protection (Figure 7b-ii).^302^ Owing to the improved charge collection and reduced recombination losses at the interface, these PSCs also had high *V*_OC_ values of up to 0.89 V. Apart from their ability to establish favorable interactions with perovskite, fullerene derivatives, such as phenyl-C_61_-butyric acid methyl ester (PCBM), can create a spike-like energy band at the top interface, leading to a strongly suppressed carrier recombination at this top interface (Figure 7b-iii).^40^ Similarly, indene-C_60_-propionic acid butyl ester (IPB) and indene-C_60_-propionic acid hexyl ester (IPH), with improved electron mobility, suppress interface recombination by providing a higher conduction band offset to hamper charge-carrier-back-transfer recombination (Figure 7b-iv).^66^

Apart from fullerenes (Figure 8), alternatives, such as carborane-based molecules, e.g., phenylamino-decorated carborane (CB-NH_2_),^273^ applied in neat Pb PSCs recently, might be worth investigating. Beyond that, the very large range of “non-fullerene acceptors”, which have been developed for organic photovoltaics, remain largely unexplored for mixed Sn−Pb perovskites.

##### 3.1.2.2 Diamines, Diammonium Salts, and Metal Doping

The results with fullerene derivatives highlight the ability of surface post-treatments to manipulate device energy diagrams, enhancing in turn the electron extraction at the top interface. In particular, compositional doping, generally realized by organic and inorganic cations that potentially incorporate/bind to the lattice or some particular amines with free electron donating pairs, at the perovskite absorber surface has proven to be an excellent strategy. Coating a layer of a neutral diamine, ethylenediamine (EDA), on the mixed Sn−Pb perovskite films leads to n-type doping of the surface (Figure 7c-i, ii),^303^ leading to a downward band bending and the passivation of undercoordinated tin at the top interface. The resultant devices have shown PCEs up to 21.74% for Br-containing mixed Sn−Pb PSCs (*E*_g_ = 1.25 eV), with a reduced voltage deficit (difference between bandgap energy and *V*_OC_) of 0.39 V. However, amines like EDA with significant basicity and high vapor pressure can also damage the perovskite films. Thus, they are in principle not ideal candidates for extensive perovskite interface engineering, especially for the Sn-based perovskite films that are generally very sensitive. In this regard, the conjugated acid of EDA, ethylenediammonium (EDA^2+^), is a better option to successfully create the n-type character of the treated film at the surface.^9^ In addition, the diammonium cation post-treatment forms a surface dipole, which facilitates electron extraction at the top surface due to the consequently enlarged built-in potential. This can suppress the interface recombination even in the device that originally displayed nonideal band alignment. One of the most attractive aspects of this strategy is its universal applicability to perovskites of different compositions (neat Pb, neat Sn, and mixed Sn−Pb), to passivate the surface through both solution-based processing and thermal evaporation (Figure 7c-iii, iv).^72^ The most pronounced improvement in device efficiency, mainly through a strong increase in *V*_OC_, is for the case of Sn-containing films, highlighting the particularly beneficial aspect of this strategy on surfaces with a high concentration of defects, which in this case are likely dominated by tin chemistry. The preferential anchoring of EDA^2+^ to Sn-related sites, i.e., V_A_(Sn), is still apparent even for wider bandgap (FA_0.8_MA_0.2_Pb_0.8_Sn_0.2_I_3_, *E*_g_ = 1.33 eV) perovskite films which contain a much lower relative Sn content.^304^ Similarly, an amino acid-based material (which will be further discussed in section 3.3 (Buried Surface)) can also be used to regulate the perovskite surface potential energy and introduce a beneficial surface dipole that facilitates electron extraction at the top surface.^305^ We note that the orientation of the ligand applied should be carefully designed. For example, in the case of cysteine,^305^ the acid group side interacts more strongly with the perovskite lattice than that of the ammonium group thanks to the assistance of the −SH group, leading to the surface dipole formed with the desired orientation (Figure 7c-i). Over the past years, diamines and diammonium salts have been extensively employed for the surface modification of p-i-n PSCs by various research groups.^144,306^ The wide structural diversity of this group of chemicals enables testing modifiers for specific desired effects. For example, by increasing the alkyl chain length, the diammonium ligand—1,3-propane-diammonium (PDA^2+^)—is more effective than EDA^2+^ in maximizing the photoluminescence quantum yield (PLQY) retention of perovskite films covered with a C_60_ layer.^307^ Despite the high similarity in the chemical structure of these two diammonium compounds, the significantly different behavior as surface modifiers points out the need for further investigation of the underlying mechanism.

While small ligands such as EDA^2+^, piperazine-1,4-diium (PP^2+^),^80^ and PDA^2+^ are unable to form a 2D perovskite on the surface of an iodide-based perovskite,^308^ increasing the chain length up to 1,4-butane-diammonium (BDA^2+^) can reconstruct the perovskite surface with a newly formed Dion−Jacobson (DJ) perovskite phase,^308^ which substantially alters the interfacial charge carrier dynamics. In general, the small diammonium ligands—EDA^2+^, PP^2+^, and PDA^2+^—dope or dedope the perovskite surface to shift the Fermi level to lie closer to the conduction band minimum (CBM), forming a more favorable electronic structure for the electron extraction at the n-type interface in p-i-n cells. The possible underlying mechanism of this doping/dedoping effect can be related to the factors that are responsible for the variation of the carrier concentrations.^309^ In this particular case, it might be caused by, for example, (i) the suppressed formation of defects raising the p-doping, tin vacancies and interstitial iodine defects,^310,311^ owing to the enhanced lattice stability; (ii) the change of the perovskite surface composition to the state with an increased formation energy of the tin vacancies;^312,313^ (iii) the change of the surface defect states being dominated by the donor-type shallow defects;^88,310^ (iv) the change of the length of the metal−halide bond and/or the tilt of the MX_6_ octahedral of the perovskite lattice,^314^ upon the binding of the ligands to the surface of the perovskite; and/or (v) the enhanced charge transfer doping from the next contact, i.e., ETL, in the stacked structure.^309,315,316^ To the best of our knowledge, however, a comprehensive study is still lacking for mixed Sn−Pb perovskites in these aspects. Meanwhile, the cyclic diamines piperazine (PP), 4-aminopiperidine (4APP), and 4-(aminomethyl)piperidine (4AMP) also lead to the formation of an n-type surface in mixed Sn−Pb perovskites.^80^ Interestingly, these diamines even react in situ with the organic material at the surface of the perovskite films, primarily FA^+^, scavenging protons from the cation(s) (Figure 7d). In these cases, the working mechanism of the diamines is likely comparable to the diammonium ligands, where molecules with less than four carbon atoms between amines/ammoniums mostly will not lead to the formation of 2D phases. However, the diamines presented additional effects caused by the mentioned deprotonation of A-site cations. On top of that, they cause more morphological “erosion” of the surface due to their stronger basicity.^80^ Alternatively, for other amines, amine-FA^+^ condensation reactions can take place, expelling ammonia, as identified for MA^+^ in solution.^317^ Methylenediammonium (MDA^2+^) also oligomerizes into hexamethylenetetramine (HMTA), which can then react with FA^+^ to form tetrahydrotriazinium (THTZ-H^+^).^82^ These reactions can potentially happen at the solid film surface, for example, when it is modified with 3-(aminomethyl)pyridine (3-APy)^61^ and likely 2-thiophenemethylamine (TMA),^318^ EDA, and other alkylamines.^50,317^ In the case of 3-APy, the product is *N*-(3-methylpyridine) formamidinium (MPyFA^+^), or for amine-MA^+^ reaction, a simple transfer or sharing of acidic protons happens, forming 3-APy^+^. The authors further claimed that the formed MPyFA^+^ cation sits at the A-site of the perovskite, with the pyridine ring promoting the formation of positively charged V_I_ (iodide vacancies), which act as shallow donors and induce a field that facilitates electron extraction. These top surface modifications seem to ensure less interfacial recombination and, accordingly, better performance for the resultant cells, but further work is necessary to fully understand multiple concurrent and unusual effects. These interesting in situ chemical reactions occurring between the neutral amines or other molecules, such as bis-diazirine,^83^ and the organic cation(s) of the precursor material offer huge potential for further exploration/improvement in PSCs and other perovskite-based applications. In particular, precisely targeted chemical reactions or surface species can be implemented into the perovskite system for specific aims.^50,318−323^ Various unexpected/underexplored chemical reactions generated in this complex system also make the perovskite research more exciting.

**Figure 9:**
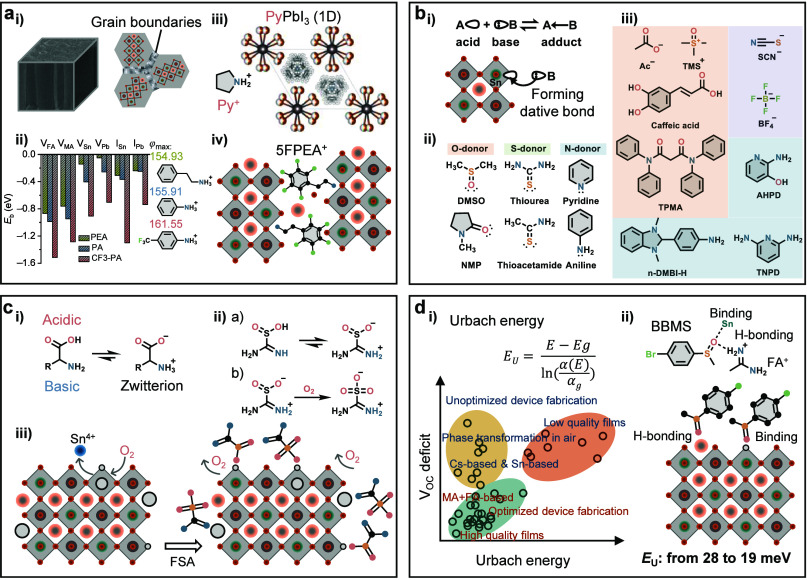
Grain boundary. (a) (i) Schematic illustration of the perovskite films and the crystallographic domain and grain boundaries. (ii) Binding energy (*E*_b_) between passivators and various acceptor-like defects. Maximum electrostatic potentials (φ) of the surfactants are provided with the chemical structure of the passivators. Reproduced with permission from ref (8). Copyright 2022 Springer Nature. (iii) Crystal structure of 1D PyPbI_3_, viewed along the *c*-axis, generated with the CIF file from ref (20). (iv) Grain boundary of mixed Sn−Pb perovskite films passivated with 5FPEA^+^. (b) (i) Lewis acid (A)−base (B) reaction to form an adduct (A·B) with a dative bond, and the Lewis base forms the dative bond with Sn(II) of the mixed Sn−Pb perovskite lattice. (ii) Lewis bases with oxygen donor (O-donor), sulfur donor (S-donor), and nitrogen donor (N-donor). Reproduced with permission from ref (33). Copyright 2016 American Chemical Society. (iii) Lewis base molecules and passivating ions that have been applied for improving the mixed Sn−Pb perovskite films. (c) (i) An amino acid contains both acidic (carboxylic acid fragment) and basic (amine fragment) centers. The isomer on the right is a zwitterion. (ii) (ii-a) Formamidine sulfinic acid (FSA) and its zwitterion. (ii-b) Oxidation of FSA. (iii) Schematic illustration of antioxidation and defect passivation at grain surfaces (including film surface and grain boundaries) of mixed Sn−Pb perovskite films enabled by FSA. Reproduced with permission from ref (44). Copyright 2020 Springer Nature. (d) (i) Equation of Urbach energy, where α(*E*) is the absorption coefficient spectra and α_g_ is the value of α at the bandgap energy (*E*_g_). Higher values of *E*_U_ show higher subgap absorption and vice versa. The relationship between device *V*_OC_ and Urbach energy shows that the value is affected by the film processing condition and perovskite composition. Reproduced with permission from ref (68). Copyright 2022 American Chemical Society. (ii) BBMS interacts with Sn(II) and FA^+^ from perovskite. BBMS binds to the mixed Sn−Pb perovskite film, leading to the Urbach energy reduction of the mixed Sn−Pb film from 28 to 19 meV. Reproduced with permission from ref (73). Copyright 2022 Wiley-VCH.

On the other hand, various metal cations have also been examined to tune the properties of mixed Sn−Pb perovskites. Bowman et al. found that the presence of Zn^2+^ ions in the precursor solution enabled improved Pb:Sn homogeneity in the as-crystalized films and increased charge carrier lifetime, but they also observed the slightly increased p-type nature of the mixed Sn−Pb perovskite.^324^ The surface of the mixed Sn−Pb perovskite films has also been improved with the small metal cation Zn^2+^, thanks to its stronger ionic interaction with the iodide than that of Sn and Pb cations.^325^ Alternatively, the perovskite lattice has also been doped with foreign metal cations by substituting the B-site cations and/or filling the B-site vacancies,^326−329^ e.g., with Cd^2+^ for mixed Sn−Pb perovskites.^330,331^ Interestingly, Huang et al. found that the alkaline earth metal cation, Ba^2+^, stays at the interstitial sites and works as a shallow electron donor, creating an n-type/less p-type surface of the mixed Sn−Pb perovskite films.^332^ This work inspires the community to study the effectiveness of the rest of the alkaline earth metal cations for improved mixed Sn−Pb perovskites, such as Mg^2+^, Ca^2+^, and Sr^2+^, bearing in mind that the perovskite material will likely be particularly sensitive to the doping concentration.^333^

The application of functionalized molecules or rationally selected metal cations on the top perovskite surface is a straightforward approach to improve charge extraction at the interface and enhance device performance. Fullerenes and, more recently, diammonium compounds and diamines have been widely employed for modifying the mixed Sn−Pb perovskite top interface, and its effectiveness has also been successfully extended to the p-i-n devices with various perovskite compositions. The easy structural tunability of these materials offers the possibility to modify perovskite surfaces with specifically desired results. Considering the vulnerable nature of the exposed surface and its importance for electron extraction, future studies should focus on strategies to precisely passivate specific defect states, especially in combination with energy level tailoring. We note that altering the surface energetic states (e.g., to a more n-type character) will likely activate halide migration, aggravating device instability.^334^ Therefore, despite the beneficial effects on the device performance of some surface passivation strategies, the detrimental side effects limit the maximum stability improvement of the resultant PSCs, especially under elevated temperatures. To overcome this trade-off, a deeper understanding of surface defect characteristics and accordingly designing improved strategies, for example, with the participation of some interesting sulfur-containing materials^335−337^ to overcome these hurdles, is of utmost importance.

### 3.2 Grain Boundaries

Perovskites processed by conventional solution-based methods form polycrystalline thin films featuring a high number of grain boundaries (Figure 9a-i). These sites contain vacancies and interstitials of different characteristics due to the intrinsic nature and instability of grain boundaries, reducing the optoelectronic quality of the film. Therefore, the passivation of grain surfaces and interfaces is critical for attaining maximum performance for solar cells. Strategies reported so far for modifying grain boundaries generally rely on introducing additives into the perovskite precursor solution. The component should be sufficiently large to avoid being incorporated into the 3D perovskite lattice, while at the same time also chemically unable to split up the 3D perovskite lattice, such that it would ultimately end up at the grain boundaries of the polycrystalline films. Additive engineering simultaneously allows for keeping tighter control of the crystallization process, with the possibility to modulate and reduce defect formation already during the thin film fabrication.

#### 3.2.1 Defect Passivation

##### 3.2.1.1 Ammonium Salts

Ammonium cations are a species that resemble the material already present in the perovskite lattice, offering a unique opportunity to use them as additives for defect passivation in grain boundaries. In addition, the structure of these molecules can be easily tuned to achieve the desired properties in perovskite thin films. The most popular additives of this family are ammonium cations,^338^ which have shown a very high reproducibility in numerous works. A comparison among PEA^+^, phenylammonium (PA^+^), and 4-trifluoromethyl-phenylammonium (CF_3_-PA^+^), using ab initio molecular dynamics simulations, suggested that these passivators are absorbed on the surface of the grain boundaries, with a binding energy that depends on its chemical nature (Figure 9a-ii).^8^ For CF_3_-PA^+^, the superior electron-withdrawing character of the fluorinated substituent results in a more electropositive ammonium head, ensuring the strongest binding with the negatively charged defects, i.e., A- and B-site vacancies. Moreover, the absorbed CF_3_-PA^+^ also reduces the donor-type defects, e.g., V_I_, by increasing the desorption energy at the corresponding sites. Although proven only for neat Pb perovskites, CF_3_-functionalized PEA surfactant (CF_3_-PEA^+^) presents a high molecular polarity, ensuring a strong interaction both with the perovskite modified underneath and the C_60_ coated atop.^339^ This enhances the charge extraction together with a reduced energetic mismatch between the perovskite and the ETL, thereby enlarging the quasi-Fermi level splitting (QFLS) and increasing the device *V*_OC_. Owing to the reduced surface defects and elongated carrier diffusion length of the mixed Sn−Pb films, the CF_3_-PA^+^ modification yielded device efficiencies of over 22% for single-junction PSCs and a certified efficiency of 26.4% for all-perovskite tandem solar cells.^339^ Varying the structure of the ammonium additives also offers the possibility to control the dimensionality of the phases that form. The medium-sized cation pyrrolidinium (Py^+^) forms a one-dimensional (1D) PySn_x_Pb_1−x_I_3_ phase (Figure 9a-iii), which passivates the film surface and grain boundaries, leading to films with elongated device operational life.^20^ Meanwhile, other fluorinated ammonium cations, such as 2,3,4,5,6-pentafluorophenethylammonium (5FPEA^+^) (Figure 9a-iv),^340^ also reduce trap-assisted recombination losses and lower the background carrier density in mixed Sn−Pb perovskite films. Overall, ammonium species are remarkably efficient for passivating the grain surfaces of perovskite films.

##### 3.2.1.2 Lewis Bases

One key species used as additives in perovskite solutions to modulate defects and control the crystallization are Lewis bases (Figure 9b-i, ii). These nucleophilic compounds can establish strong interactions with perovskite precursor materials during film processing and inside the final films (Figure 9b-iii).^33,341^ For instance, the antioxidant caffeic acid has the ability to prevent Sn(IV) formation, modulate the crystallization, and mitigate the defect densities in the films.^86^ The molecule owes its versatile properties to the presence of the Lewis base carboxylate and the antioxidative hydroxyl groups. As a result, the solar cells presented a *V*_OC_ of 0.855 V with enhanced shelf stability. Likewise, *N*,*N*,*N*′,*N*′-tetraphenylmalondiamide (TPMA), a β-diketone-based Lewis base ligand, can effectively passivate the undercoordinated Pb(II) defects and interact with Sn(II) in perovskite films to inhibit its oxidation via binding with the ketone group.^342^ In addition, the hydrophobic benzene groups ensure an improved resistance of the films. Apart from various donor groups from organic species,^343−347^ the coordination with the metal can also be realized by compounds bearing pseudohalide anions, such as thiocyanate (SCN^−^),^247^ acetate (Ac^−^),^348^ and tetrafluoroborate (BF_4_^−^).^242^ For instance, the addition of KSCN improves the resistance of Sn(II) against oxidation, since the SCN^−^ anions interact with the undercoordinated Sn(II). These modified mixed Sn−Pb PSCs show enhanced air stability, with reported solar cells maintaining over 50% of their initial PCE after 5 days of air exposure.^349^ Moreover, K^+^ cations can reduce grain boundaries and defect states in films.^350^ Thus, the proper design of inorganic/organic salts offers the possibility to introduce both cations and anions with different benefits simultaneously. For example, imidazolium tetrafluoroborate (IMBF_4_) contains the IM^+^ cation that passivates the surface and its counteranion BF_4_^−^ that reduces the lattice strain of the films.^351^ As a result, the mixed Sn−Pb films show improved quality and reduced structural disorder.

**Figure 10:**
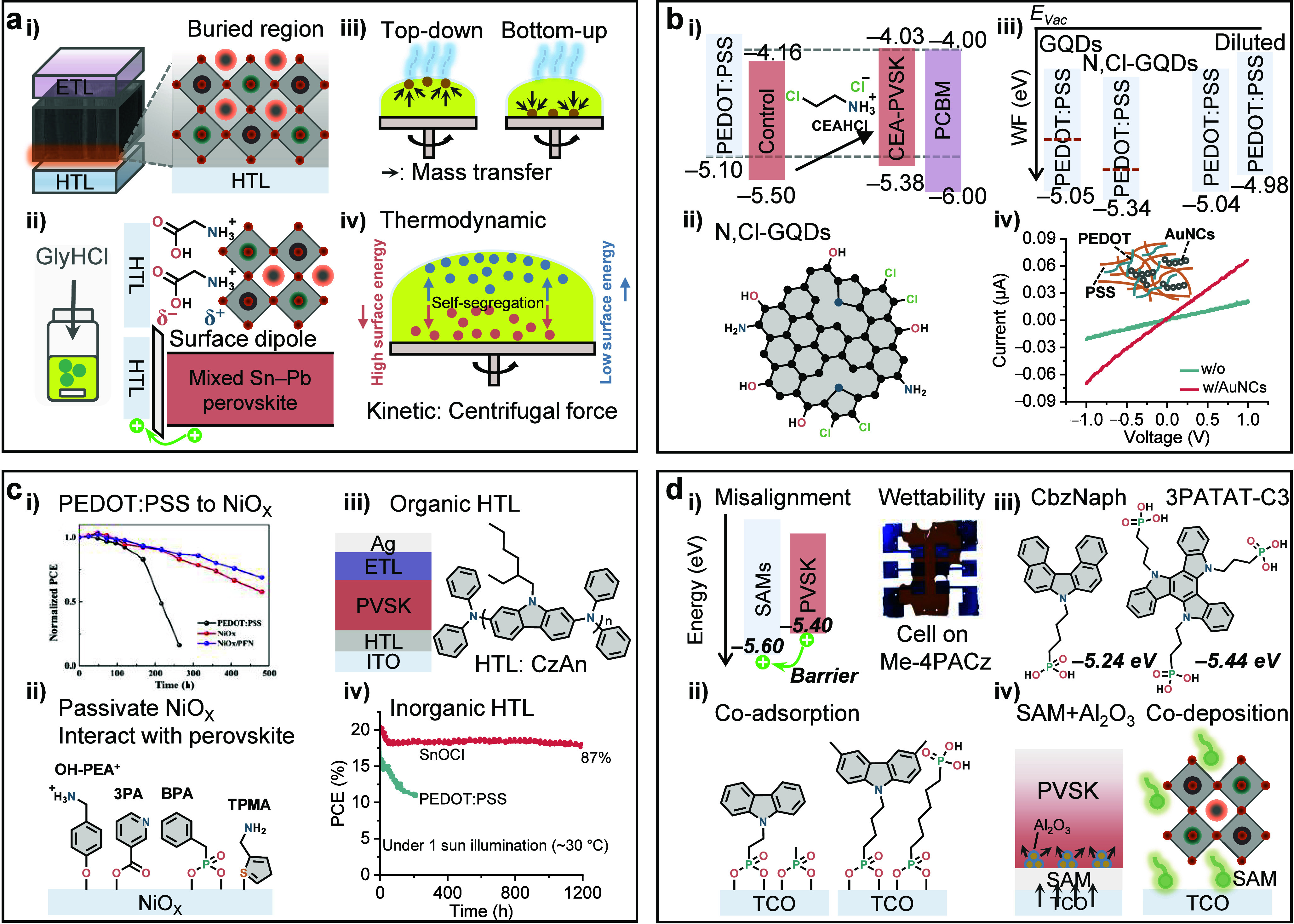
Buried interface. (a) (i) Schematic illustration of the perovskite films sandwiched with the charge transport layer with the buried interface highlighted. (ii) Schematic illustration of GlyHCl as an additive for processing mixed Sn−Pb perovskite films, creating a surface dipole at the buried interface by the preferential molecular accumulation. (iii) Schematic illustration of top-down and bottom-up crystallization. (iv) Schematic for the mechanism of additive segregation during the film formation. Reproduced with permission from ref (11). Copyright 2023 Springer Nature under a Creative Commons Attribution 4.0 International License. (b) (i) Tuning the energy level of the perovskite with CEAHCl treatment. Reproduced with permission from ref (25). Copyright 2022 American Chemical Society. (ii) Structure of N,Cl-codoped graphene quantum dots (GQDs). Reproduced with permission from ref (35). Copyright 2022 Elsevier. (iii) Tuning the energy level of PEDOT:PSS by GQDs doping or dilution. (iv) Current−voltage (*I*−*V*) characteristics of the PEDOT:PSS films without and with Au nanochains (AuNCs) modification. The plot was generated with WebPlotDigitizer 4.6 based on the original graph published.^43^ Copyright 2022 Royal Society of Chemistry. (c) (i) Long-term stability of the mixed Sn−Pb PSCs with different HTMs. Reproduced with permission from ref (69). Copyright 2021 Wiley-VCH. (ii) Molecules bind to the NiO_X_ HTL to passivate its defects, alter its work function, and/or passivate the defects in the perovskite films. (iii) New organic HTL drawing CzAn as an example. (iv) New inorganic HTL presenting the device stability of SnOCl modified HTL as an example. The plot was generated with WebPlotDigitizer 4.6 based on the original graph published in ref (74). Copyright 2022 Wiley-VCH. (d) (i) Schematic illustration of the misaligned energy levels of the perovskite and SAM-based hole selective contact (left). Optical images of the finished devices, showing the substrate coverage by triple-cation perovskite films fabricated on the Me-4PACz selective contact layer (right). Reproduced with permission from ref (75). Copyright 2023 American Chemical Society under CC-BY 4.0. (ii) Co-adsorption of SAM and small filler, MPA (left) and 6dPA (right). (iii) Chemical structures of CbzNaPh and 3PATAT-C3. The correspondingly modified ITO substrates show HOMO levels of −5.24 and −5.44 eV, respectively. (iv) Schematic illustration of SAM and Al_2_O_3_-based hole selective contact (left). Schematic illustration of the SAM molecule codeposited with perovskite precursor solution (right).

##### 3.2.1.3 Zwitterions

Zwitterionic molecules are another promising family of additive candidates for achieving substantial passivation at surfaces and grain boundaries, as they contain an equal number of positively and negatively charged binding groups (Figure 9c-i). As an example of a small zwitterionic compound, formamidine sulfinic acid (FSA)^44^ reduces both the donor- and acceptor-like defects via binding to the sites with its sulfinic and acetamidinium heads, respectively. Apart from the defect passivation effect, FSA can also suppress the formation of Sn(IV) at the precursor solution, thanks to its reducing ability (Figure 9c-ii, iii). Thus, multifunctional additives offer multiple opportunities for processing high-quality mixed Sn−Pb perovskite films and efficient solar cell devices.

#### 3.2.2 Energy Disorder

Another potential of the additive engineering strategy is the reduction of energy disorder. The energetic disorder of the perovskite films is generally evaluated with the Urbach energy (*E*_U_), determined from the absorption tail of the films. Most lead halide perovskite polycrystalline films have an *E*_U_ of around 12 to 20 meV,^352^ with Sn containing perovskites generally having *E*_U_ over 20 meV,^68^ with the absolute value related to the device processing or nature of the perovskite composition (Figure 9d-i). This indicates the existence of subgap defects, which contribute to the Urbach tail and act as Shockley−Read−Hall recombination centers. Therefore, lower *E*_U_ values in films should imply that these samples also have longer charge carrier diffusion lengths and carrier lifetimes. For mixed Sn−Pb perovskite, the addition of 1-bromo-4-(methylsulfinyl)benzene (BBMS) and SnF_2_ leads to a record-low *E*_U_ of 19 meV.^73^ The films showed improved crystallinity and resistance to oxidation, and consequently, the treated devices presented outstanding stability, retaining 98% of their original efficiency after heating at 60 °C for 2660 h under N_2_. BBMS enables less defective grain surfaces and interfaces via its ability to interact with the organic and inorganic cations, i.e., FA^+^ and Sn^2+^ (Figure 9d-ii).

The defects at the surfaces are closely related to the degradation processes in devices, and therefore, their mitigation is key to achieving long-term stable PSCs. This fragile and defective nature of grain boundaries can be easily abated by employing the proper molecules as additives in the precursor solution during the thin film formation process. The functionalities present in these compounds will determine their ability to bind the perovskite materials, affecting the crystallization process and being able to bind with specific defect sites. Lewis bases and ammonium salts are the most widely studied additives for processing Sn-containing perovskite films with higher quality, fewer defects, and higher resistance against external stimuli. They benefit from their excellent ability to not just modulate the crystallization dynamics but also bind with the readily oxidized Sn^2+^ and mobile ions at the grain surfaces. Thus, developing functional molecules that improve the grain surfaces is essential for achieving high-quality polycrystalline perovskite films. Meanwhile, grain-boundary-free films, i.e., single-crystalline perovskite films,^81,353^ would also be a direction worth exploring for these materials. In any case, a deeper knowledge and control over the crystallization process of neat Sn and mixed Sn−Pb perovskites remains a necessity, which will open the door to the fabrication of materials with improved intrinsic quality and reduce the dependence on such a high number of additives/impurities.

### 3.3 Buried Surface

Efficient charge carrier management at the buried surface is critical for proper device functioning. This can be achieved by modifying the perovskite film and/or adjacent HTL (Figure 10a-i).^354^ Generally, a reduced energy-level offset between the HTL and the perovskite layer would provide benefits for the hole extraction and at the same time reduce the nonradiative charge carrier recombination at the interface. Also, this interface is typically most strongly affected by defects, mainly due to imperfect crystallization of the perovskite material or the detrimental chemical reactions occurring at the bottom surface. Lowering the defect density at the interface would minimize surface recombination and alleviate material degradation, thus improving the stability of the solar cell devices.

#### 3.3.1 Amino Acid Salts

To address the imperfections at the buried interface, the addition of amino acid salts, e.g., glycine hydrochloride (GlyHCl), into the precursor solution of mixed Sn−Pb perovskites is one of the most effective strategies (Figure 10a-ii).^9^ Based on the NMR study, the GlyH^+^ cation preferentially binds to the perovskite colloidal particles at the early stages of the processing, due to chemical interactions of the ammonium head with the octahedral units of the perovskite colloids in the solution. This binding causes the particles to reach relatively larger sizes and consequently be heavier than the free component in the solution. During the following crystallization processes, the heavy particles sediment on the substrate, leading to the accumulation of GlyH^+^ at the bottom region of the as-prepared films. In particular, the ammonium head of GlyH^+^ binds to the perovskite lattice primarily due to the −NH_3_^+^ group dominating the interaction of the molecule with perovskites over −COOH,^9^ and the electronegative carboxyl groups at the bottom surface face outward from the perovskite, toward the HTL. This results in a surface dipole at the buried interface, which creates an electric field that assists in driving the holes to the HTL. Besides this facilitated hole extraction at the buried surface, GlyHCl also increases the crystallinity and reduces the defect density of the films. As a result, the mixed Sn−Pb PSCs (*E*_g_ = 1.25 eV) resulting from this strategy achieved a PCE of 23.6%, with an FF of 0.82 and a *V*_OC_ of 0.91 V (∼93% of the radiative limit). The unencapsulated devices also showed improved stability under AM 1.5G, retaining over 80% of the initial efficiency after 200 h under continuous maximum power point tracking in an inert atmosphere at ∼55 °C. This GlyH^+^ additive strategy has since been successfully adopted by several other groups working with mixed Sn−Pb PSCs and all-perovskite tandem cells.^355,307,356−359^

The successful and targeted introduction of the additives in the perovskite films requires a profound understanding of the crystallization mechanism. Regarding the crystallization direction of the films, the downward (top-down) pathway generally applies more frequently than the upward (bottom-up) path for the films processed via solution-based methods (Figure 10a-iii). In most cases, this happens since the evaporation of the residual solvent is initialized from the top surface of the “wet” films, leading to the advanced supersaturation and the formation of nuclei.^360^ The downward crystallization can, however, lead to fluctuating distributions of the additive based on its properties. In a downward crystallization process, generally, the additives, if not volatile, will be locked at the top surface when their colloidal particles have low solubility.^360,361^ Meanwhile, if colloidal particles show a higher solubility and weigh more than the colloids containing only 3D perovskite precursors, they will be sedimented at the bottom region of the wet films.^9^ Exceptionally, if the additives establish no strong interaction with the perovskite or contain an ion that is exchangeable with the perovskite composition,^348^ they will most likely be squeezed out and end up accumulating at the bottom region of the films. In some specific cases,^362−364^ however, bottom-up crystallization dominates. For example, keeping the surface of the films wet and intentionally exposing them to solvent vapor would allow the crystal growth to initialize from the buried interface.^365^ By manipulating the compositions of the intermediate states, interestingly, multiple crystallization routes can be present. For example, in perovskite (MAPbI_3_), perovskite/MA_2_Pb_3_I_8_(DMSO)_2_, and MA_2_Pb_3_I_8_(DMSO)_2_, perovskite crystal growth can take place through a downward-growth, both downward- and upward-growth, and upward-growth mechanisms, respectively.^366^ The effect in the mixed Sn−Pb perovskite system is yet to be examined, however. Furthermore, if significant chemical interactions exist between the ligand and substrate, this will also lead to the accumulation of the additive at the bottom interface.^367^ Recently, Wang et al.^11^ claimed that the centrifugal force (kinetics), together with the unfavorable enthalpic interactions (thermodynamics) between additive and perovskite components, provides a strong driving force for the additive segregation in the final spin-coated films (Figure 10a-iv). Thermodynamic considerations dictate that high-surface-energy polar moieties tend to migrate toward the substrate, i.e., downward segregation, to minimize the free energy of the system.^368^ Conversely, nonpolar low-surface-energy moieties preferentially enrich the top surface to reduce the overall free energy of the system, by substituting high-surface-energy perovskite components at the surface, i.e., upward segregation. We note that the knowledge established in solution-based processes may not be directly transferable to vacuum-based dry processing as the crystallization routes are mostly different.^369^

Back to amino acid salts, the ones with long chain lengths, such as 5-aminopentanoic acid (5AVA^+^),^370,371^ will, however, induce the formation of low-dimensional perovskites.^370,372^ Using amino acids with a length no larger than 3-ammonium propionic acid (3APA^+^) would avoid the formation of low-dimensional phases as the appropriate spatial conformation.^372^ Additives with long chain lengths, on the other hand, will largely confine the perovskite growth and result in a more remarkable reduction of grain size compared to their shorter chain counterparts.^373,374^ More research on the solution chemistry and the new intermediate states^154,375^ that form due to the different amino acid salts,^376^ especially using in situ measurements,^377^ will guide the design of new materials and the optimization of the cell fabrication processes. However, a global picture of how these molecules affect the precursor solution stage and how this impacts the crystallization process, including their influence on defect states and carrier dynamics in cells, is mostly lacking.

#### 3.3.2 Interlayers and PEDOT:PSS Modifications

Currently, the main advancements of efficient mixed Sn−Pb PSCs largely use PEDOT:PSS as the HTL. This is mainly because of the following: (i) The superior wettability of the PEDOT:PSS film^378^ provides sufficient nucleation sites for the rapidly crystallized Sn-containing perovskite films with no pin holes.^379^ (ii) The well optimized PEDOT:PSS film processing protocol guarantees the fabrication of compact films with high reproducibility, thus reducing current leakage in the device and improving the yield of efficient PSCs. (iii) The redox-inactive nature, under normal conditions, of PEDOT:PSS reduces the possibility of the Sn(II) oxidation of the Sn-containing perovskites. However, maximizing the potential of PEDOT:PSS requires sometimes various modifications at the bottom interface. For example, managing the energy level of perovskite films can provide a better alignment with the HOMO level of the HTL, suppressing carrier nonradiative recombination at the buried interface. To this end, a layer of 2-chloroethylammonium (CEA^+^) coated between the perovskite films and the HTL can reduce defect densities and enhance the antioxidative character at both the surface and the bulk of perovskite films (Figure 10b-i).^25^ As a consequence, solar cell efficiency and air stability increased, with over 50% PCE retained by unencapsulated devices after 400 h in ambient air. Meanwhile, the use of ammonium salts which contain extra functional groups, such as carboxylic and halide/pseudohalide species, is another promising and versatile additive strategy for mixed Sn−Pb perovskite systems.

Other strategies for the treatment of the bottom contact focus on inserting a p-type layer between perovskite and PEDOT:PSS films, or modifying PEDOT:PSS itself to achieve a more favorable energy level alignment (Figure 10b-ii, iii),^380^ a reduced interfacial resistance,^381^ and a further enhanced conductivity, e.g., by gold nanochains (AuNCs) (Figure 10b-iv).^43^ For instance, graphene quantum dots (GQDs)^35^ can be easily functionalized with electron-deficient atoms to alter their work functions and give them a p-type character. Accordingly, the N,Cl-codoped quantum dots (N,Cl-GQDs) induce an improved band alignment with the perovskite films, achieving mixed Sn−Pb PSCs (*E*_g_ = 1.25 eV) with improved stability, an efficiency of 21.5%, and a *V*_OC_ of 0.89 V. On the other hand, simultaneously introducing cations and anions at the buried interface through their salts can generate some synergistic effects on both the PEDOT:PSS and the perovskite layers. It was found that incorporating potassium citrate, a weak base, into the PEDOT:PSS can not only neutralize the acidity of PEDOT:PSS but also improve the quality of the perovskites thanks to the coordination of citrate anion with the Sn(II) centers and the potassium cation enhanced film crystallinity.^382^ The resultant mixed Sn−Pb PSCs show a PCE of up to 22.7% with a *V*_OC_ value of 0.894 V, together with improved stability.

#### 3.3.3 Other HTL Modifications

Nevertheless, the long-term stability of mixed Sn−Pb PSCs is largely restricted due to the acidic and hygroscopic nature of the conventional bottom contact, the PEDOT:PSS layer.^383^ Finding suitable substitutes is thus critical for ensuring the intrinsic stability of the cell stack.^384^ NiO_X_ is the most studied HTL, with the ability to increase the stability of the PSCs compared to the PEDOT:PSS-based devices (Figure 10c-i), maintaining about 91% of its original efficiency at 80 °C for 20 h and 92% of its initial performance after 46 days storage in inert conditions.^385^ However, NiO_X_ presents Ni^≥3+^ sites and multiple types defects that can induce degradation pathways at the perovskite interface.^386^ The insertion of poly[(9,9-bis(3′-(*N*,*N*-dimethylamino)propyl)-2,7-fluorene)-*alt*-2,7-(9,9-dioctylfluorene)] (PFN) successfully passivates these sites, as well as improving the energy level alignment.^69^ With this strategy, PSCs with a high *V*_OC_ of 0.88 V and outstanding stability for the encapsulated cells under ambient conditions can be fabricated. Meanwhile, small polar molecules, such as 4-hydroxyphenethylammonium (OH-PEA^+^),^387^ have also been applied to reduce the defect states of NiO_X_ films and improve the energy level alignment at the buried interface of the PSCs. In these cases, the surfactants generally interact/react with NiO_X_ through the terminal that can bind with the metal center, e.g., −OH,^387^ −COOH,^388^ −PO(OH)_2_,^386^ −S,^389^ etc., to passivate the defects in the NiO_X_ layer (Figure 10c-ii). Then, the opposite terminal of the surfactant points away from the NiO_X_ and to the perovskite material coated above, altering the work function of NiO_X_ and concurrently passivating the defect states of the perovskite layer. In addition, this strategy could also abate the delamination-induced failure of solar cell devices, thanks to the enhanced interlayer interaction.^390^ Interestingly, tuning the work function of NiO_X_ can also be realized by modifying its processing conditions, such as the annealing temperature.^391^ In comparison with high-temperature-annealed NiO_X_ films, the films processed at room temperature show improved crystallinity and reduced Ni vacancies, leading to a deeper valence band and lower trap densities.^391^

Despite the evident advantages of the intrinsic chemical properties of NiO_X_ versus PEDOT:PSS, the processed films are generally less conductive and not chemically inert^298^ and defect-free.^386^ Thus, the community is still constantly looking for some alternative promising organic and inorganic HTLs (Figure 10c-iii, iv)^384,392−395^ and hole-selective self-assembled monolayers (SAMs),^396,397^ or the best combination of these two. A solution-processed ternary tin(II) alloy (SnOCl) has been proposed as a novel HTL for mixed Sn−Pb PSCs.^74^ Due to its textured structure, SnOCl layers provide reduced optical losses in the full device stack. In addition, it induces a well-controlled grain growth with the suppressed formation of small grains at the buried interface. The resultant PSCs presented greatly enhanced stability (87% of their initial efficiency retained after 1-sun illumination for 1200 h and 85% under 85 °C thermal stress for 1500 h). Superior efficiencies of 23.2 and 26.3% for the single-junction devices and the all-perovskite tandem cells, respectively, were realized with a mixture of SnOCl and neutral PEDOT, thanks to the improvement in the coverage and work function alignment with the indium tin oxide (ITO) substrate.

The application of SAMs in Sn-containing perovskites has faced many challenges that have delayed the first reports of their successful implementation, in comparison to the first ones in neat Pb PSCs (Figure 10d-i).^398,399^ However, when 2-(9*H*-carbazol-9-yl)ethyl]phosphonic acid (2PACz) was blended with methyl phosphonic acid (MPA), mixed Sn−Pb PSCs reached an efficiency of 23.3% and a significantly improved stability, i.e., no loss after 1000 h of constant illumination under inert conditions.^400^ Besides matching the energy levels, here, the key to achieving efficient PSCs fabricated on SAM-based FTO substrates is related to improving their coverage through the MPA filler (Figure 10d-ii). FTO substrates used are generally textured, which can lead to current leakage and consequently energy loss at the interface. Due to negligible parasitic absorption, SAM-based cells show a large current gain compared to their PEDOT:PSS-based counterparts. Compared with the neat Pb PSCs, however, we find that the SAM-based mixed Sn−Pb PSCs do generally suffer from reduced batch-to-batch reproducibility. This problem has led to very few successful attempts made by a limited number of groups in the Sn-containing PSCs.^400−402^ There are several reasons related to this, e.g., (i) unsuitable energy level alignment which will generate severe nonradiative carrier recombination, and (ii) poor coverage of the SAMs on the TCO substrate that will lead to the current leaking and/or detrimental side reactions between the perovskite and substrate. Most of the SAMs developed currently have HOMO levels around −5.6 to −5.9 eV,^396,403^ which match the valence band maximum (VBM) of the neat Pb perovskite films but is much deeper than the VBM of most Sn-containing analogues, generally from −5.0 to −5.4 eV,^12,379,404,405^ as the energy level of the Sn 5s orbital is shallower than that of 6s of Pb.^172^ Therefore, tuning the work function of the SAM-based contact by introducing small binding molecules that can—to some extent—compensate for the polarity of the employed SAM is worth pursuing. Furthermore, the development of new SAMs with energetics adjusted to Sn-containing perovskite films is important (Figure 10d-iii).^406^ We note that the TCO substrates covered with Me-4PACz^403^ SAM show exceptionally poor wettability to the perovskite precursor solution, even for the neat Pb-based perovskites with a DMF-rich solvent system (Figure 10d-i).^75^ Al_2_O_3_ nanoparticles^63,407,408^ and/or some ammonium salts^409^ as a wetting layer, or 1,6-hexylenediphosphonic acid (6dPA) as a second component to the SAM precursor solution,^75^ can be implemented to overcome this wettability issue, enabling solar cells with improved efficiency and reproducibility as well as alleviated film delamination. Thus, an improved understanding of SAM processing, especially the impact from the processing solvent, and the surface chemistry, alongside the design of new versatile^410^ and processing-tolerant SAMs, will be key to aiding the community to reach the next milestone on both cell efficiency and reliability.^144^ To further simplify the PSCs processing, codeposition of the hole-selective contact and the perovskite absorber would be worth investigating in future work. Taking advantage of the chemical interactions between the bottom bare FTO/ITO substrate and SAMs, SAM molecules introduced directly from the precursor solution system would also guarantee the device with good performance (Figure 10d-iv).^411−413^ This strategy would also allow for alteration of the energy level of the perovskite films at the bottom region, creating a bend banding at the buried interface that benefits hole extraction.^413^

Obtaining efficient PSCs without any hole transport/selective contact at all should also be feasible once the energy levels are well aligned in the heterojunction,^207^ e.g., realized by some particular treatments,^414^ and there are no detrimental reactions, e.g., Sn(IV) oxidation,^415^ taking place at the interface. Mixed Sn−Pb PSCs stacks without HTL generally present elongated operation lifetimes compared to conventional PEDOT:PSS-based PSCs. HTL-free PSCs capped with a sputtered indium zinc oxide (IZO) electrode are currently one of the most stable mixed Sn−Pb PSCs reported.^207^ These PSCs retained 95% of their initial efficiency after 1000 h, at 85 °C in the air under dark conditions without encapsulation as well as in a damp heat test with encapsulation. Under operating conditions (0.8 sun was used), the ITO-sandwiched cells with no metal top contact fully maintained their initial efficiency for over 1000 h under inert conditions. This indicates that removing defects and degrading materials at transport layers can greatly improve the performance of the PSCs.

**Figure 11:**
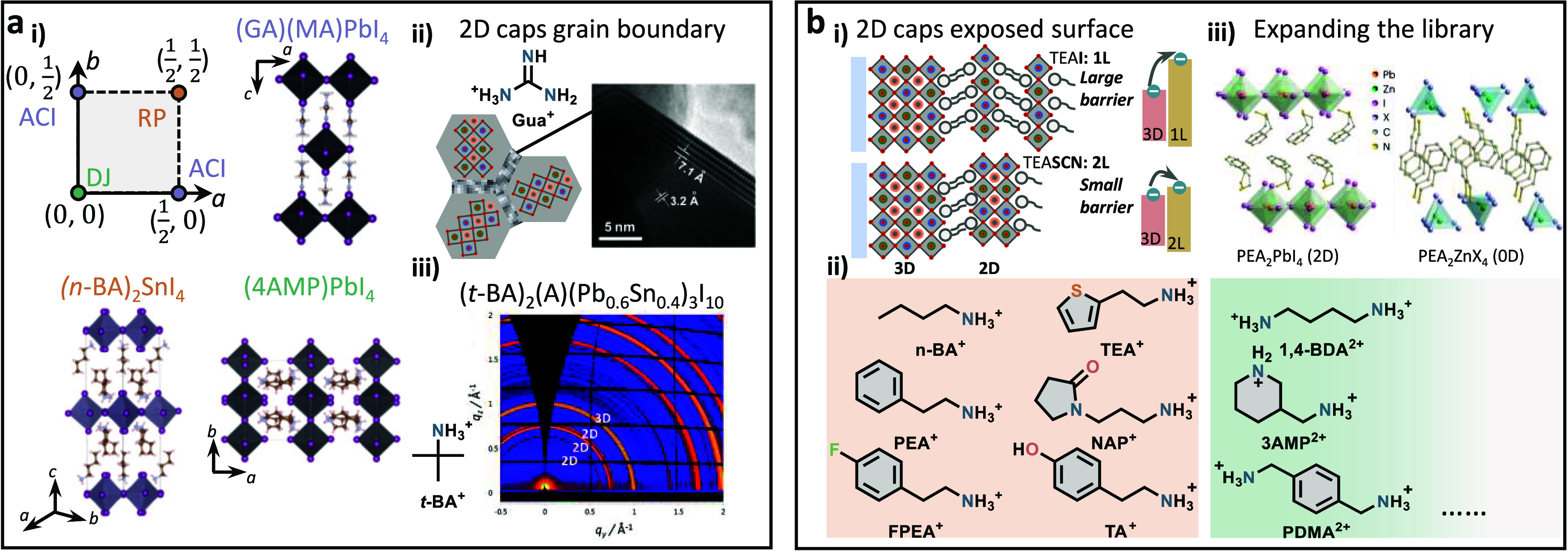
2D capping. (a) (i) Schematic illustration of the 2D perovskite phase space with boundaries defined by the ACI, RP, and DJ crystal phases (corresponding to alternating cations, Ruddlesden−Popper, and Dion−Jacobson phases, respectively). The coordinates indicate the shift between consecutive perovskite layers along the *a* and *b* axes of the 2D perovskite structure. (0, 0) and (1/2, 1/2) correspond to the eclipsed and staggered structures. Crystal structures of (GA)(MA)PbI_4_,^1^ (*n*-BA)_2_SnI_4_,^2,3^ and (4AMP)PbI_4_^10^ shown as an example of the structures of ACI, RP, and DJ perovskite phases, respectively. (ii) Schematic illustration of 2D-capped grain boundaries of the mixed Sn−Pb perovskite films along with the HRTEM image of the grain boundary region of the perovskite prepared with 7% GuaSCN additive. Reproduced with permission from ref (29). Copyright 2019 American Association for the Advancement of Science. (iii) 2D GIWAXS patterns for (*t*-BA)_2_(A)(Pb_0.6_Sn_0.4_)_3_I_10_ (A = FA_0.85_Cs_0.15_) films. Reproduced with permission from ref (36). Copyright 2018 American Chemical Society. (b) (i) Schematic illustration of mixed Sn−Pb film surface capped with different low-dimensional phases. The 2L phase formed with the TEASCN treatment shows a lower interfacial energetic barrier than the 1L phase generated with the TEAI treatment. (ii) Molecular structures of the spacers used for modifying mixed Sn−Pb perovskites. (iii) Expanding the low-dimensional perovskite phase library from the Pb-based to the Zn or other metal-based new materials. Reproduced with permission from ref (51). Copyright 2023 Springer Nature.

Different strategies can be employed to improve the buried perovskite surface. Modification or replacement of conventional PEDOT:PSS can avoid the instability issues linked to it. In particular, SAM-based and HTL-free structures recently showed high potential for enhancing device efficiency and stability. On the other hand, we noticed the delamination of perovskite films from SAM-based substrates during the high-temperature annealing, e.g., at 150 °C. This can—to some extent—be alleviated by using a thin substrate or preheating the substrate and/or using the SAM molecules that could interact with or chemically bind to the perovskite atop. From a different perspective, the additive GlyHCl proved the ability of specifically functionalized molecules to form large perovskite colloids and primarily sediment on the bottom surface to facilitate charge extraction. Similarly, amino acids or SAMs with −PO(OH)_2_ or −COOH acid terminals can not only passivate the defects in NiO_X_ and perovskite films but also induce an intentional modification of the bottom region of the films, tuning the energy level and facilitating the hole extraction. These works point out the broad applicability of amino acids for improving the bottom interface in mixed Sn−Pb PSCs, which we believe has a strong potential yet to be uncovered, in terms of structural and mechanistic diversity. Besides the top surface modification mentioned above, Al_2_O_3_ nanoparticles show excellent ability to solve the wettability issue for different substrates and passivating the buried interface. This also indicates that modifying both the exposed and buried surfaces of perovskite films using Al_2_O_3_ nanoparticles will boost the performance of the solar cells further. Ideally, a similar function could also be provided by the other analogues, such as SiO_2_ and ZrO_2_. We note that the dielectric nature of these nanoparticles will likely cause FF reduction of the cells because of the series resistance loss when increasing the thickness of the interlayer. Thus, a porous insulator contact design^63^ was recently developed to mitigate this trade-off effect. These new functionalities will allow the community to target more efficient and robust device structures, particularly with simplified procedures^412^ to be easily implemented into large areas and flexible cells.^81^

### 3.4 2D Capping

3D perovskites generally stack with infinite corner-sharing octahedral units, while 2D perovskites form when they cleave along a crystallographic plane, e.g., ⟨100⟩, ⟨110⟩, or ⟨111⟩, to form sheets that are linked with large cations. The properties of 2D perovskites can be easily tuned by changing the layer thickness (defined by the n number), the cage cation, and the spacer cation, resulting in excellent structural diversity. The most common 2D perovskites are the ⟨100⟩-oriented ones, which can be further divided into the alternating cations in the interlayer space (ACI) phase,^1^ Ruddlesden−Popper (RP) phase,^416^ and Dion−Jacobson (DJ) phase (Figure 11a-i).^10^ In the different phases, the inorganic slabs of the 2D perovskites are defined as quantum wells, while the spacer acts as a barrier.^308^ Due to the quantum and dielectric confinement effects,^417^ 2D perovskites present interesting semiconductor characteristics, such as an increase in bandgap with the decrease of the 2D layer thickness, and the tunability of the exciton binding energy by the dielectric constant of the spacer cation. Due to their high structural formation energy and the hydrophobic nature of the spacer, 2D perovskites also show excellent stability under different stimuli, such as heat and humidity. However, these 2D phases generally display low carrier mobility, which induces current loss in the devices. As we noted in the above sections, therefore, judicious design is required when using the 2D spaces to cap the grain boundaries and surfaces of the 3D polycrystalline perovskites being employed to fabricate efficient and stable photovoltaics.

For mixed Sn−Pb PSCs, the most widely implemented spacers are guanidinium (GA^+^) (ACI-type) and PEA^+^ (RP-type), while the DJ-type has been relatively less explored. The ACI-type spacer GA^+^ can be used to form 2D-composed grain boundaries with suppressed tin vacancies and enhanced structural stability (Figure 11a-ii).^29^ These 2D-capped 3D films show a largely reduced energetic disorder, increased carrier lifetimes, and reduced surface recombination velocity. The resulting mixed Sn−Pb perovskite (*E*_g_ = 1.25 eV) single-junction cells reached > 20% efficiency, as well as 25% for 4-T and 23.1% for 2-T all-perovskite tandem cells. Several groups have since successfully applied GA^+^ cations to enhance the performance of PSCs.^418−420^ Apart from the potential passivation effects from the three ammonium groups, the authors also claimed that GA^+^ cation also offers the possibility of tuning the band structures, generally moving the Fermi level of the perovskite closer to the CBM, due to the reduced background hole density.

An RP-type 2D or quasi-2D Sn−Pb perovskite, (*t*-BA)_2_(FA_0.85_Cs_0.15_)_n−1_(Pb_0.6_Sn_0.4_)_n_I_3n+1_ (n = 2−9, *t*-BA: *t*-butylammonium) was first investigated in 2018 for its application in solar cells (Figure 11a-iii).^36^ The authors found that perovskites composed of n = 4 2D species display superior ambient stability, presumably owing to the combined suppression of both inherent defects as well as externally (air) induced degradation. Later on, the PEA^+^ cation was extensively examined for its exceptional ability to regulate crystal growth and suppress multiple defect states.^281,421−423^ In addition, a fluorinated PEA^+^ cation, 2-(4-fluorophenyl)ethylammonium (FPEA^+^), is effective for regulating the 2D/3D mixed phases, causing film formation with a preferential crystal orientation perpendicular to the substrate plane.^424^ Unencapsulated mixed Sn−Pb PSCs with FPEA^+^ capping showed enhanced stability under both inert and ambient conditions. Aiming to maximize the gain from 2D species, dual spacer cations were also proposed, because of their synergistic complementary effects on the manipulation of crystallization and carrier transport.^425,426^ Combining previously discussed PEA^+^ and GA^+^ spacers,^29^ a preferential formation of the n = 2 PEA_2_GAPb_2_I_7_ phase can be induced into the grain surface and the 3D film surface.^426^ In comparison to the n = 1 pure 2D structure, n = 2 quasi-2D structures present longer carrier lifetimes and better out-of-plane charge transport. Thus, it enables minimizing the charge recombination and enhancing the charge extraction at the 3D/2D interface. As a result, mixed Sn−Pb PSCs reached PCE values of 22.3% with outstanding *V*_OC_ values as high as 0.916 V, representing the best values reported for perovskite absorbers with a bandgap of ∼1.25 eV. At the same time, tandem solar cells showed PCE values of up to 25.5%. More importantly, the solar cells presented improved operation stabilities, with over 82% of their efficiency maintained after 1830 h in N_2_ for the mixed Sn−Pb single-junction cells, and with 80% of the initial efficiency maintained after 1500 h operation in N_2_ for the 2-T tandem cells. Interestingly, when applying 2-thiopheneethylammonium on top of the as-prepared films,^427^ a 2L quasi-2D structure can be formed by using an SCN-based salt, which is different from the 1L 2D phase generated with the I-based salts (Figure 11b-i). Thanks to the reduced energy barrier at the top interface, the carrier transfer and the performance of the cells fabricated with the 2L capped film were substantially improved compared with the 1L case. Other 2D phases composed with new spacers have also been investigated as capping layers for the 3D bulk perovskites, such as *N*-(3-aminopropyl)-2-pyrrolidinone and 4-hydroxyphenethylammonium,^428^ and the DJ types, *p*-phenyl dimethylammonium,^429^ 3-(aminomethyl)piperidinium,^430^ 3,4-dihydroxyphenethylammonium,^287^ and 1,4-butanediammonium diiodide (Figure 11b-ii).^431^

The capping of 3D perovskite films with 2D perovskite phases is a key strategy for achieving perovskite films with improved quality and stability. In addition, the modulation of the spacer and layer number of the 2D phase allows for fine-tuning of the properties of this layer. Future work should focus on understanding the role that the low-dimensional species play in altering the semiconductor properties of the novel mixed-dimensional perovskite films (Figure 11b-iii).^51^ DJ-type species would generally be expected to be more stable than RP-type species, due to the elimination of the weak van der Waals forces between the spacers. However, the stability of DJ phase perovskites also largely relies on the property of the spacer utilized. For example, the DJ phase composed with the spacer 1,4-cyclohexanedimethylammonium (CyDMA^2+^), with medium rigidity, is more robust to the external stimuli than the DJ phase composed with a spacer having higher or lower rigidity, e.g., phenylenedimethylammonium (PhDMA^2+^) and hexyldiammonium (HDA^2+^), respectively.^432^ For low-dimensional phases, however, similarly to MA^+^ cation,^433^ the ammonium spacers may eventually suffer from thermally induced chemical decomposition or reorganization in films.^434^ Therefore, more studies on 2D species, especially the DJ type, are needed to achieve more robust mixed dimensional Sn−Pb PSCs. Finally, mixing the spacers^435^ to balance the pros and cons of each of them would also be a very promising way to improve the mixed Sn−Pb perovskites for photovoltaic applications.

### 3.5 Section Summary

Surfaces in perovskite films are vulnerable sites prone to high defect densities and degradation. Surface modification strategies have proven critical to improving the quality of mixed Sn−Pb perovskite films in p-i-n solar cells. Depending on the specific requirements of each perovskite and device composition, surface treatments can be adapted to selectively improve a certain perovskite surface: (i) The top perovskite surface can be substantially improved with fullerene derivatives and diammonium/diamine molecules, where the bonds established with surface defects play a key role. (ii) Grain boundaries can be modified with functional additives like Lewis bases and ammonium species that can modulate crystallization and passivate defects at the grain surfaces. (iii) For the buried interface, the conventional HTM, PEDOT:PSS, can be modified or substituted with, for example, efficient SAMs, or novel functional molecules as additives that modify perovskite colloids and accordingly the buried interface. (iv) Easily tunable 2D phases can be applied to cap the 3D perovskite domains to enhance their optoelectronic properties and stability profoundly. The knowledge gained through the works discussed here should motivate the community to develop novel passivating agents and device structures that successfully limit surface defects throughout the perovskite films. In parallel, a deeper understanding of the perovskite crystallization process and related defect generation and defect nature would allow better control of the final film quality. The development of enhanced passivation strategies for perovskite surfaces in the future will allow a critical advancement in the efficiency and stability of mixed Sn−Pb PSCs, pushing the field forward toward commercialization of efficient all-perovskite tandem photovoltaics.

## 4 All-Perovskite Tandems

**Figure 12:**
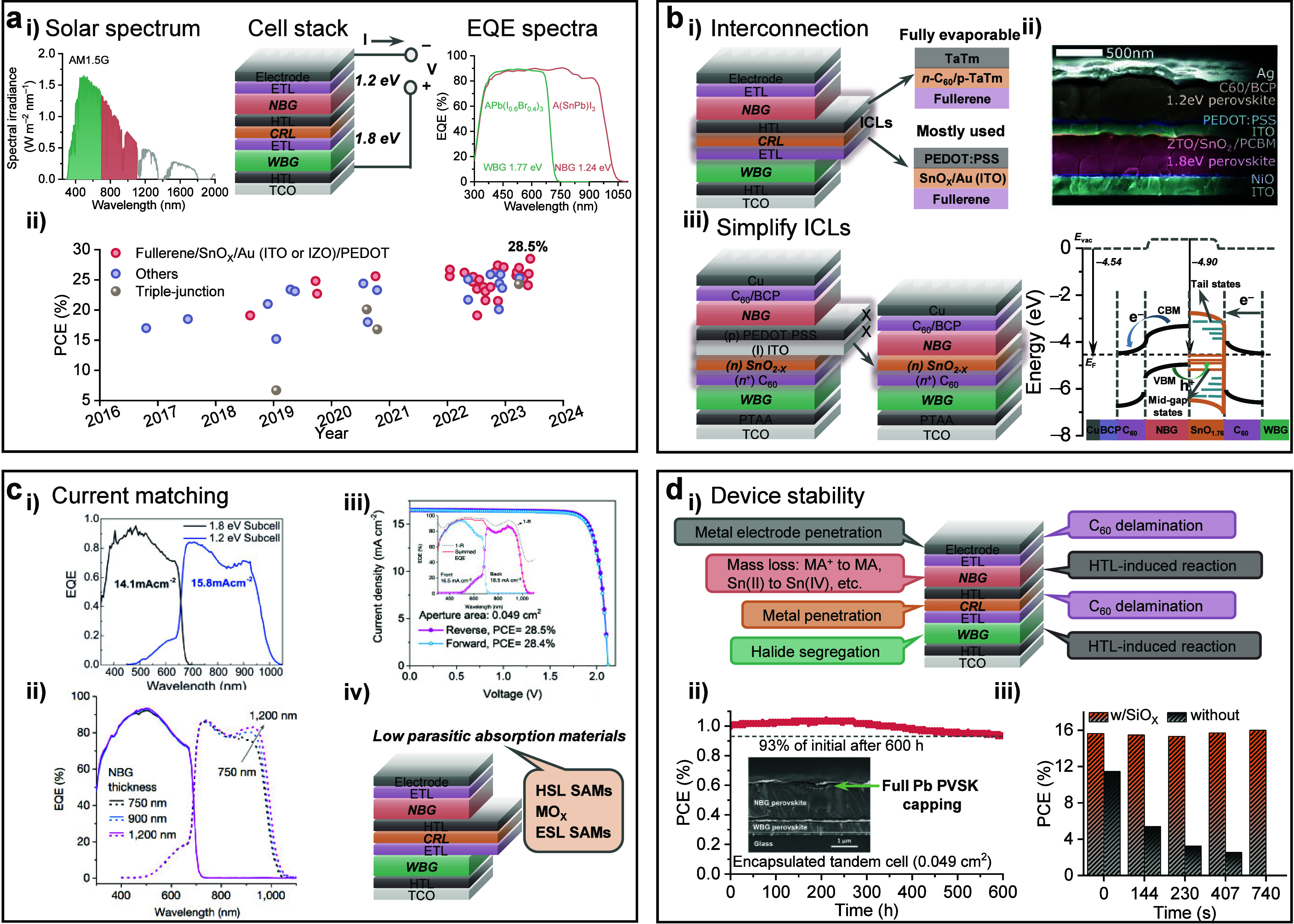
Tandem cells. (a) (i) ASTM G173-03 reference solar spectrum. The data was downloaded from NREL. Cell stack of monolithic double-junction two-terminal all-perovskite tandem solar cells and EQE spectra of the WBG and NBG subcells. The EQE spectrum of the NBG cells was generated with the data published before in ref (9). Copyright 2022 Royal Society of Chemistry. Meanwhile, the EQE spectrum of WBG is unpublished data from our lab. (ii) Efficiency progress of mixed Sn−Pb perovskite-containing all-perovskite tandem solar cells, updated by June 10, 2023. (b) (i) Schematic illustration of the tandem cell stack highlighting the interconnecting layers. The most used interconnection layers have the structure of fullerene/SnO_X_/Au (ITO)/PEDOT:PSS based on the reported cell data plotted in Figure 12a-ii. (ii) Scanning electron micrograph of a 2T perovskite−perovskite tandem. Reproduced with permission from ref (31). Copyright 2016 American Association for the Advancement of Science. (iii) Schematic illustration of simplifying the interconnecting layers by removing the ITO and PEDOT:PSS layers (left). Energy diagram for the C_60_/SnO_1.76_/NBG/C_60_ layers in all-perovskite tandem solar cells (the full device structure is shown at the bottom) (right). The energy diagram shows that the holes from the NBG perovskite film are injected into the SnO_1.76_ through the midgap states (orange lines) and then recombine with electrons extracted from the WBG perovskite film by the doped C_60_ layer. *E*_vac_ and *E*_F_ denote vacuum and Fermi levels, respectively. Reproduced with permission from ref (41). Copyright 2020 Springer Nature. (c) (i) EQE spectra for the 1.8 and 1.2 eV subcells. Reproduced with permission from ref (31). Copyright 2016 American Association for the Advancement of Science. (ii) EQE spectra of the best CF_3_-PA device (with 1.2-μm-thick absorber). Reproduced with permission from ref (8). Copyright 2022 Springer Nature. (iii) *J*−*V*, EQE, and total absorptance (1-R) curves of the champion tandem device with perovskite heterojunction. Reproduced with permission from ref (77). Copyright 2023 Springer Nature. (iv) Schematic illustration of the tandem cell stack highlighting the application of low parasitic absorption materials, such as the SAM-based hole selective layer (HSL) and electron selective layer (ESL), and MO_X_ (M = metal) layer. (d) (i) Schematic illustration of the tandem cell stack highlighting the main origin of the stability loss from each device layer. (ii) Continuous maximum power point (MPP) tracking of an encapsulated tandem solar cell over 600 h under simulated AM 1.5G illumination (100 mW cm^−2^, multicolor LED simulator) in ambient air with a humidity of 30−50%. The device had an initial PCE of 27.4%. The device temperature was around 35 °C during operation due to the self-heating under solar illumination. There was no passive cooling during device operation while the environmental temperature was kept at around 25 °C. The plot was regenerated by WebPlotDigitizer 4.6 based on the original graph published in ref (77). Copyright 2023 Springer Nature. Inset shows a cross-sectional SEM image of all-perovskite tandem solar cells highlighting the full lead perovskite capped at the top of the NBG mixed Sn−Pb perovskite films. Reproduced with permission from ref (77). Copyright 2023 Springer Nature. (iii) Bar graphs showing the PCEs of mixed Sn−Pb devices measured in the ambient without (black) and with (orange) SiO_X_ for various ambient exposure times. Reproduced with permission from ref (97). Copyright 2023 Springer Nature.

Tandem technologies are one of the most promising applications for metal halide perovskite materials (Figure 12a).^24,27,34,37,436,437^ Nevertheless, the challenges that all-perovskite tandems face are still plenty.^38,438^ First, the interconnection layer should have optimal properties to allow holes and electrons from both cells to recombine efficiently. However, the deposition of high-quality layers is complicated and could potentially damage the perovskite material underneath. Second, maximizing the current matching of the two absorbers is not trivial, as the bandgap, thickness, and crystal quality of both perovskite films have to be perfectly optimized for the particular tandem cells. Third, all-perovskite tandems suffer from poor stability largely inherent to mixed Sn−Pb perovskite subcells,^439^ caused by, for example, the thermally and photochemically unstable MA^+^ content, the oxidation of Sn(II), and the acidity and hygroscopicity of the often used PEDOT:PSS HTL. Finally, the deposition of all of the layers and the upscaling of the process is a complex task that requires careful optimization. In this section, we summarize the limitations of current protocols, the challenges faced by the field, and future promising research directions to enable efficient and stable all-perovskite tandem solar cells.

### 4.1 Interconnect

To connect the subcells of the tandem devices, the interconnection layers (ICLs) are indispensable (Figure 12b-i). The CRL acts as a medium that collects holes and electrons from the interfaced subcells and allows them to recombine with each other. An efficient interconnecting layer largely determines the shape of the devices’ current density−voltage (*J*−*V*) characteristics, which should concurrently possess low contact resistance, high optical transparency, and mechanical/chemical robustness. Finding a perfect candidate is, however, very challenging.

For constructing the device, the interconnecting layer is generally composed of ETL(HTL)/conducting layer/HTL(ETL) where the conducting layer is optional occasionally. For instance, in n-i-p tandems, doping the HTL with Li-TFSI and tBP additives greatly improves the efficiency of the tandems stacked with no extra conducting layer.^440^ The Li/Li^+^ redox shuttle takes the holes and electrons from the subcells to be involved in the reaction, improving the conductivity of the interconnecting layer. The dopant, however, can potentially react with the perovskite materials with the migration of the small ions, e.g., Li^+^, accelerating the device’s degradation or failure. Inert materials are highly preferred in this regard. For example, the less mobile, 2,2′-(perfluoronaphthalene-2,6-diylidene (F_6_-TCNNQ) dimalononitrile) and *N*^1^,*N*^4^-bis(tri(*p*-tolyl)phosphoranylidene)benzene-1,4-diamine (PhIm) dopant are used to dope *N*^4^,*N*^4^,*N*^4′′^,*N*^4′′^-tetra([1,1′-biphenyl]-4-yl)-[1,1′:4′,1′′-terphenyl]-4,4′′-diamine (TaTm) and C_60_, respectively, serving as the extra charge selective layer between the half cells in the p-i-n tandem architecture.^441,442^ In addition, these dopants can be deposited with a vacuum-based thermal deposition method, avoiding the air exposure of the subcell fabricated at first and the use of the solvent that is generally harmful to the environment. These doped extra layers, however, likely cause a considerable increase in the fabrication cost of tandem photovoltaics and introduce additional parasitic absorption/detrimental interfacial carrier recombination that limits the efficiency of photon conversion.

Thanks to the low production cost, low absorption, and high stability, ALD-grown thin, while compact, layers of metal oxide materials, such as Al_2_O_3_, ZnO, SnO_2_, and TiO_2_, are broadly implemented (Figure 12b-ii) to reduce the damage to the front perovskite absorber from the solvent-mediated processing of the second subabsorber.^443^ To increase the ohmic contact, extra material(s) are then deposited above the metal oxide(s), e.g., metal Ag or Au (generally of about 1 nm thickness and hence forming noncontinuous nanoparticles),^8,426,444,445^ or sputtered-ITO,^31,446^ indium zinc oxide (IZO),^397^ and aluminum-doped zinc oxide (AZO).^84,447^ However, these materials commonly introduce parasitic absorption that increases with the thickness, and the metals potentially penetrate through the layers and react with the perovskite material;^448,449^ moreover, additional layers also increase the device processing complexity. Accordingly, they cause cost increases, loss in the energy-lifetime yield, and longevity reduction of the tandem PVs. Thus, a metal-free CRL is recommended.^41,450^ The direct contact of ALD-SnO_2_ and PEDOT:PSS (in p-i-n structure) generally leads to s-kinks in the *J*−*V* curves, due to the formation of a Schottky barrier. To overcome this, it is pivotal to control the properties of the deposited metal oxide and develop bifunctional layers that allow direct contact with the perovskite.^446^ A single layer would need to collect both electrons and holes from opposite sides; therefore, the ambipolar carrier transport property is required. To this end, increasing the defect density of the ALD-SnO_X_ would help to improve the carrier density and thus conductivity of the layer (Figure 12b-iii). Lowering the content of Sn(IV) in SnO_X_ can accordingly increase the density of Sn(II) in the layer, which induces middle-gap energetic states that attract charge carriers.^41^ In addition, intentionally creating defect states by controlling the pulse length of the SnO_X_ precursors—e.g., tetrakis(dimethylamino)tin and deionized water^450^—allows for fabricating layers with desired carrier concentration and sufficiently low contact resistance.

From the chemistry aspect, there is still much room for modifying the properties of the ALD-SnO_X_ layer. Searching for new superior MO_X_ (M = metal) layers, or developing nucleation media that allow growing the CRL conformally and efficiently, is a critical step for the further improvement of tandem photovoltaics, specifically for flexible devices.^84^ The interconnecting layer also affects the output voltage of the tandem cells. It is potentially caused by the detrimental interfacial recombination generated at the perovskite interface contacts with ETL/HTL. In principle, the approaches that reduce interfacial recombination in single-junction cells will also be effective in tandem devices sharing the same contact.^451^ Overall, a simple yet effective charge recombination architecture for advancing the performance of all-perovskite tandem devices is highly demanded. So far, the ICLs have been largely limited with very thin (ALD-SnO_2_/Au/PEDOT) or wrong refractive index (ITO) materials. Developing conductive, red and near IR transparent and higher refractive index interlayers is an important future direction.

### 4.2 Current Matching

In the monolithic configuration, where all subcells are connected in series, the current through the different subcells is ideally the same, while their voltages are added. Therefore, good matching of the current generated by the two subcells is crucial for maximizing their operating performance.

In general, the output current of PSCs is largely associated with the bandgap of the perovskite absorber. As vastly examined, the ideal match of the subcell in monolithic double-junction two-terminal tandem PVs is ∼1.2 and ∼1.8 eV absorbers for the NBG rear and WBG front cells, respectively (Figure 12c-i).^31,452^ The bandgap increases with the amount of I^−^ substituted by Br^−^ ion at the X-site of the neat Pb perovskite films,^453^ owing to the influence of the anion electronic states.^454^ For the WBG subcells, thus, the composition with the I/Br ratio around 3/2 gives the neat Pb perovskite with a bandgap of ∼1.8 eV.^453^ Meanwhile, the single accessible way currently to lower the bandgap down to ∼1.2 eV is the B-site substitution, i.e., a certain amount of Pb(II) replaced by Sn(II) cation, thanks to the anomalous bandgap bowing effect.^236^ To reach a bandgap as small as ∼1.2 eV, the Sn/Pb ratio would be close to 1/1, as given by most of the reports.^31,251,455^

With the ideally matched bandgaps, maximizing the output current still requires further optimization. For example, it can sometimes be realized by optimizing the thickness of the subabsorbers. Reducing the thickness of the WBG absorber would allow more midenergy photons to escape from the front subcell and accordingly be absorbed by the rear subcell. As for the NBG absorber, a thickness over 1000 nm would be required to generate the current that matches the current generated from the WBG subcell (Figure 12c-ii, iii). However, increasing the thickness of the NBG mixed Sn−Pb perovskite films generally leads to lower performances, due to the insufficient carrier lifetime and diffusion length. Therefore, strategies to improve the current match by enhancing the quality of thick mixed Sn−Pb perovskite films have been extensively investigated.^8,29,185,330,332,426,452,456^ Usually, the big grain size in polycrystalline films indicates good perovskite quality and, thus, long carrier lifetime and diffusion length as well. Some strategies to improve the quality of the films involve retarding the crystallization of the films by, for example, slowing down the release speed of the solvent from the wet intermediate-phase perovskite films^31,456^ or introducing halide or pseudohalide ions in the process.^29,457,458^ However, films with big grain sizes do not always give high output current of the cell, as it also associates with their crystallinity and orientation. Films with <100>-dominated orientation generally show higher carrier mobility; thus, manipulating the crystal growth to make the film oriented with the face possessing the highest carrier mobility is also equally critical. Generally, this can be realized by changing the processing method^459^ and/or introducing some specific additives^154,305^ or low-dimensional spacers^459,460^ to regulate the crystal growth. As we introduced above, in addition, the unintentional p-doping caused by the Sn(II) oxidation and abundant defect states raised by the imperfect surfaces also shorten the carrier lifetime of the films even with big and well-oriented grains. Using antioxidants,^44^ reducing agents^185^ or ionic dopants^330,332^ to alleviate the p-doping and surfactants^8^ to passivate the surface imperfections would effectively elongate the carrier lifetime of the mixed Sn−Pb perovskite films, consequently eliminating the current mismatch of the tandems. Alternatively, light trapping engineering would also contribute to extending the light absorption for the rear subcell. For example, embedding a light-scattering micrometer-sized particle layer into a perovskite to trap light effectively increases absorptance in the infrared region.^42^ Composing the subcells with the films having big grains, desired orientation, elongated carrier lifetimes, and extended light traveling path would be beneficial for the current matching in tandems.

From the CRL side, materials with low absorbance would allow more photons to penetrate through and get absorbed by the rear NBG absorber (Figure 12c-iv), thus refining the current match of the resultant tandem devices. For instance, the absorption coefficient of poly(3-hexylthiophene-2,5-diyl) (P3HT) is higher than that of poly[bis(4-phenyl)(2,4,6-trimethylphenyl)amine] (PTAA) in the visible light range;^440,461^ thus, it will decrease the number of the photons absorbed by the rear subcell of the corresponding n-i-p tandem cells. Currently, the most employed CRL is composed of ALD-SnO_2_ sandwiched charge selective layers. Given the p-i-n structure as an example, C_60_ or PCBM/SnO_2_/Au or ITO or IZO/PEDOT:PSS is the most tested combination (refs (8, 15, 32, 44, 70, 90, 185, 305, 307, 330, 332, 339, 397, 444−446, 456, 458, 460, 462−466)) even for the multijunction cells.^93,101^ Further simplifying the structure will reduce the parasitic absorption of the CRL layer as we discussed in section 4.1, and accordingly, ameliorate the current match in tandems. As for the charge selective layers, we need to search for more transparent alternatives, even though the absorption of C_60_ and PCBM is mainly in the UV−vis region where a multi-junction cells could be affected.^467^ In this regard, we anticipate a huge potential of using hole/electron selective SAMs or evaporable fullerene derivatives with binding moiety^468^ as this will also possibly solve the delamination issue caused by the weak interaction between the perovskite and charge selective layers, especially the C_60_ layer.^469^ However, compared with the hole-selective SAMs, electron-selective SAMs are less developed.^449^ In addition, hole-selective SAMs that allow the fabrication of the Sn-containing PSCs with high efficiency and reproducibility are still largely missing because of various reasons we discussed above (with more details that we recently summarized^144^). This would ask for more efforts from the community, especially from the chemistry aspect. On the other hand, an extra gain on the output current could be realized by properly introducing antireflection materials out of the cell^70,470^ or even out of the encapsulation glass.^471^ Additionally, moving from two-terminal to four-terminal tandems^452,472^ would face no current mismatch issues but would imply needing to manufacture two separate tandem modules and then to laminate them together.

### 4.3 Device Stability

Stability is key to realizing the practical application of efficient perovskite photovoltaics. As for all-perovskite tandems, the quality of every layer matters to the operational life of the devices (Figure 12d-i). In this section, we mainly discuss the subcells in all-perovskite tandems, while leaving the rest of the discussion on stability to already published focus reviews.^37,473−475^

The operational stability of both the WBG and NBG perovskite subcells still requires significant improvement for these tandems to become commercially viable. The WBG subcells often suffer from halide segregation, which leads to increased charge transport and open-circuit voltage losses, resulting in lower PCEs.^476^ Strategies such as compositional, interface, and additive engineering have been successfully employed in an effort to increase the operational stability of these WBG subcells.^477,478^ Furthermore, thermally induced phase control has been shown to reduce defects and prevent halide segregation, leading to strongly improved device stability.^479^ Finally, the use of ionic liquids has been demonstrated to effectively stabilize the perovskite phase and make it less susceptible to environmental stresses.^447,480^ We herein also suggest the readers take a look at the focus reviews on the stability of WBG subcells published recently.^481^

The evident loss in the durability of the all-perovskite tandems largely comes from the severe MA^+^ reliance of the perovskite composition, especially in the NBG subcell. In many studies, a mixed Sn−Pb perovskite film with good quality generally requires the MA^+^ content to be no less than 30% of the A-site component.^12,29,146,185^ Involving MA^+^ cation in the A-site improves the crystallinity of the films, while its volatile nature recedes durability,^482^ especially under thermal stress^210,483^ and light soaking.^484^ Lowering the MA^+^ content while maintaining the mixed Sn−Pb perovskite films with superior quality is challenging, however, as the crystallization process is relatively hard to control compared to the neat Pb analogues. For example, decreasing the MA^+^ cation down to 10% will imply changing the fabrication process with the addition of a crystallization modulator to maintain/improve the film quality.^459^ In the MA-free system,^154,228,446,485^ the PCE of the cells is generally lower than the films containing MA^+^ cation.^9,281,400^ Interestingly, the addition of Rb^+^ cation in the MA-free mixed Sn−Pb perovskite films seems to effectively increase the film quality in terms of defect density and carrier lifetime.^228,486^ The origin behind this is, however, yet to be fully understood, for example, from the view of solution chemistry, since increasing the content of the inorganics most likely varies the colloidal properties of the precursor solution. We believe that the MA-free mixed Sn−Pb PSCs will ultimately outperform the MA-containing counterparts, while more understanding of the crystallization and the solution chemistry is required. Moreover, PEDOT:PSS also induces instability due to its notorious acidity and hygroscopicity.^383^ A PEDOT:PSS-free structure,^207^ and using modified PEDOT^42,465,487^ or all-SAM based structure,^488^ should be explored in all-perovskite tandems. Therefore, we think that, with the combination of optimal cell structure and dimensional and surface engineering for the films,^29,44,426,459,485^ the MA-free system with environmentally friendly processing protocols will dominate future studies. These aspects can also be applied to the stability enhancement of the WBG neat Pb subcells in tandem devices.^32,339,397,445,462,466,489^ Based on the literature, the currently reported record efficiency for monolithic all-perovskite tandem cells is 28.5%, and more importantly, the cells showed promising stability as well, with 93% initial PCE being retained after 600 h MPP (maximum power point) tracking under simulated one-sun illumination at around 35 °C (Figure 12d-ii).^77^

Under ambient conditions, on the other hand, stability loss mainly happens due to the loss of Sn-based content. Therefore, developing in situ and ex situ encapsulation techniques is highly important (Figure 12d-iii). Room temperature nondestructive encapsulation^490^ that allows PSCs to endure the damp heat and high-temperature light soaking test would be the long-standing pursuit. Changing the cell structure from the conventional superstrate to the substrate configuration together with depositing the mixed Sn−Pb perovskite films beforehand would allow the robust neat Pb films to act as an in situ encapsulant. As proved by Wang et al., this kind of tandem can even endure air exposure for up to hundreds of hours.^70^ The ex situ encapsulation is even more crucial considering its ability to improve the robustness of the whole device.^491^ Interestingly, it was found that the tandems implemented in space show high proton irradiation robustness,^213^ suggesting its promising potential for satellite and space exploration applications.^492−494^ Before that though, the community still needs to develop new robust materials/techniques that would guarantee the device to be able to sustain atomic oxygen, high vacuum, and large temperature variations.^97^

The stability of a mixed Sn−Pb single junction and the corresponding tandem cells under operational conditions is lagging when compared to the sharp efficiency increase, restraining their further development and application.^495^ This requires further study to understand the degradation mechanism of mixed Sn−Pb perovskites and the influence of the perovskite composition and the device architecture, especially in the tandem device.

### 4.4 Trends and Different Device Architectures

Besides standard, solution-processed two- or four-terminal all-perovskite tandem solar cells, which currently dominate the field, there are also developments beyond these standard devices, in terms of both deposition methods and different device architectures. An overview of recent attempts can be found below.

#### 4.4.1 Deposition Methods and Modularization

As for single-junction PSCs, one of the major challenges for all-perovskite multijunction solar cells lies in the upscaling of device fabrication. The fabrication of commercially viable perovskite multijunction cells requires deposition techniques that can provide a homogeneous film formation at high throughput rates. To tackle this issue, several approaches have been applied, with varying success rates.

**Figure 13:**
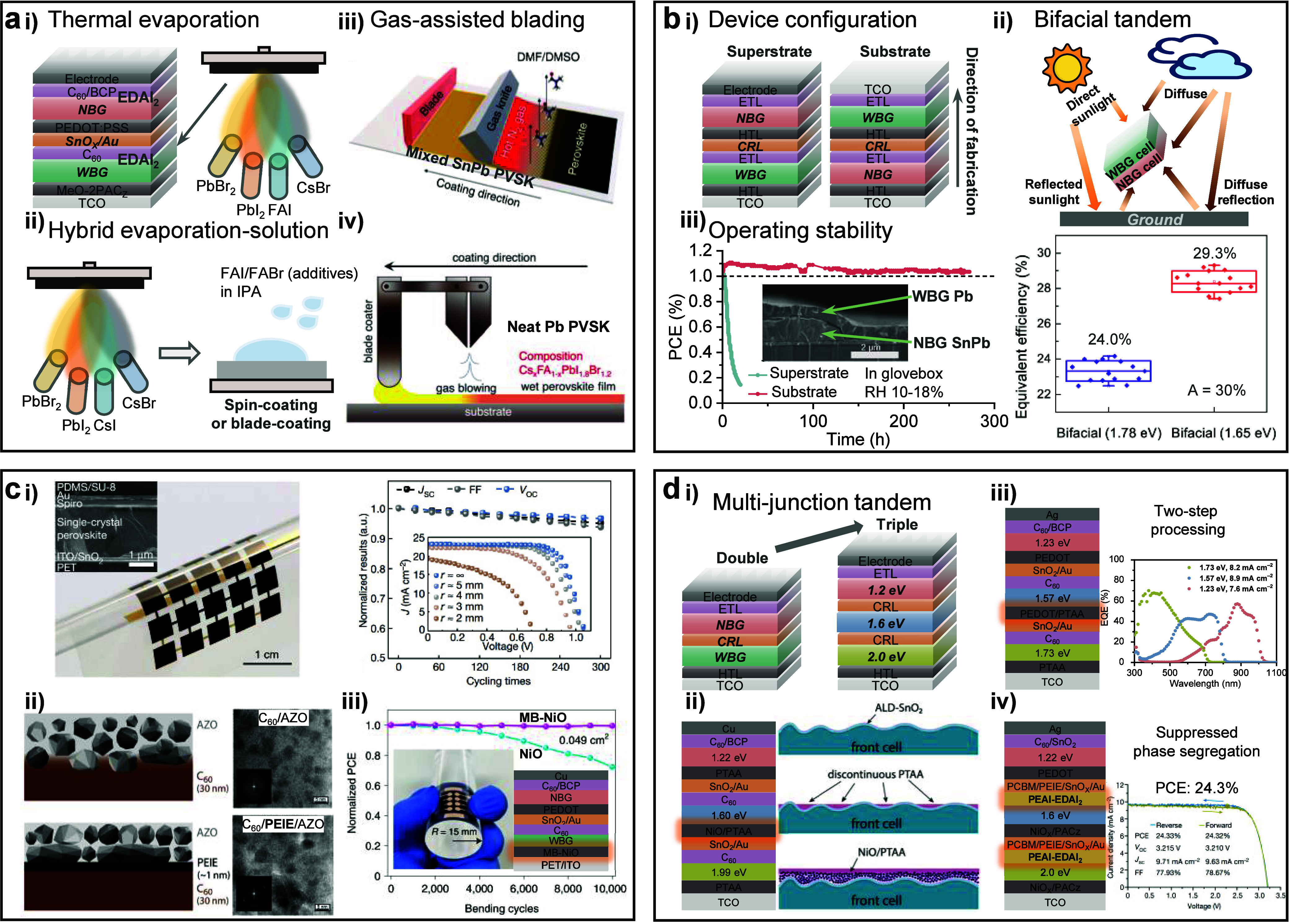
Trends and different device architectures. (a) (i) Schematic illustration of the architecture of the all-perovskite tandem solar cell with both WBG and NBG subabsorbers fabricated with the EDAI_2_ post-treatment. The WBG front absorber was fabricated with thermal evaporation.^5^ (ii) Schematic illustration of hybrid evaporation-solution fabrication route with the inorganic and organic materials deposited by the vacuum-based thermal evaporation and solution-based spin coating or blade coating. (iii) Schematic of hot gas-assisted blade coating applied for the NBG mixed Sn−Pb perovskite subabsorber fabrication. Reproduced with permission from ref (15). Copyright 2022 Springer Nature. (iv) Schematic of hot gas-assisted blade coating applied for the WBG neat Pb perovskite subabsorber fabrication. Reproduced with permission from ref (32). Copyright 2022 American Association for the Advancement of Science. (b) (i) Schematic illustration of the superstrate and substrate cell configuration. (ii) Sketch of light absorption in a bifacial all-perovskite tandem device with additional albedo light (top). Equivalent efficiency distribution of 15 bifacial tandems fabricated in the same batch with 1.78 and 1.65 eV WBG top cells under illumination with 30% of albedo light (bottom). Reproduced with permission from ref (42). Copyright 2022 American Association for the Advancement of Science under a Creative Commons Attribution NonCommercial License 4.0 (CC BY-NC). (iii) Operating stability of unencapsulated superstrate- and substrate-configured tandems. The encapsulated device retained 100% of its initial efficiency after 600 h of operation. All tests were carried out under 1-sun illumination (100 mW cm^−2^) and maximum power point tracking. The plot was generated by WebPlotDigitizer 4.6 based on the original graph published in ref (70). Copyright 2023 Springer Nature under a Creative Commons Attribution 4.0 International License. Inset: Cross-sectional SEM image of the edge of a substrate-configured tandem. Reproduced with permission from ref (70). Copyright 2023 Springer Nature under a Creative Commons Attribution 4.0 International License. (c) (i) Optical image showing an array of flexible single-crystal photovoltaic islands with a total working area of 6.25 cm^2^ (0.5 cm × 0.5 cm × 25). Inset: Cross-sectional SEM image of the single-crystal perovskite photovoltaic device (left). Cycling test results of the graded photovoltaic device at r ≈ 5 mm. Inset: *J*−*V* curves at different bending radii (right). Reproduced with permission from ref (81). Copyright 2020 Springer Nature. (ii) Schematic depicting AZO growth on C_60_ and PEIE-treated C_60_ surfaces. Transmission electron microscopy images of 5 nm C_60_/4 nm AZO and 5 nm C_60_/PEIE/4 nm AZO showing differences in AZO structure at the C_60_ interface with fast Fourier transform insets to highlight differences in crystallinity. Reproduced with permission from ref (84). Copyright 2019 Elsevier. (iii) Bending tests of flexible tandem cells based on NiO and MB-NiO with a bending radius of 15 mm. The initial PCEs of flexible tandem cells based on NiO and MB-NiO are 22.0% and 24.6%, respectively. Inset: Digital image and device structure of a flexible tandem cell under bending. Reproduced with permission from ref (90). Copyright 2022 Springer Nature. (d) (i) Schematic illustration of the device stack showing the double- and triple-junction tandems. (ii) Device configuration of 1.99 eV/1.60 eV double-junction cell and schematic diagram of PTAA and NiO/PTAA layers spin coated on the front subcell. Reproduced with permission from ref (93). Copyright 2020 American Chemical Society. (iii) Triple-junction device configuration and EQE spectra of 1.73, 1.57, and 1.23 eV subcells in a triple-junction device with C_60_/SALD-SnO_2_/Au/PEDOT:PSS ICLs. The *J*_SC_ was obtained by integrating with the AM 1.5G spectrum. Reproduced with permission from ref (100). Copyright 2020 Springer Nature under a Creative Commons Attribution 4.0 International License. (iv) Schematic diagram of device structure and *J*−*V* curves of reverse and forward scans for champion all-perovskite triple-junction tandem cells. Reproduced with permission from ref (101). Copyright 2023 Springer Nature.

Evaporated perovskite multijunctions might be the most promising candidate for upscaling, with a recent report of record efficiency of 24.1% for two-terminal all-perovskite tandem cells with vacuum-deposited WBG perovskite films (Figure 13a-i).^5^ The NBG perovskite films, however, were deposited from the solution-based process. Several attempts have been previously made to deposit mixed Sn−Pb perovskites through vacuum deposition,^258,496,497^ with the ultimate goal to fabricate all-perovskite multijunction solar cells where both subcells are deposited through vacuum-based deposition methods. However, the quality of the mixed Sn−Pb perovskite films still needs to be improved, with bulk recombination currently being one of the major limiting factors. This is likely because, on the one hand, the route has less ability to allow for maximizing the effect of the key additives, such as SnF_2_, and, on the other hand, controlling the perovskite stoichiometric composition as precisely as in solution-based methods is increasingly challenging. The difference in the physicochemical properties of the organic and inorganic perovskite precursor materials causes severe crosstalk of the materials in the codeposition procedure. Thus, like for solution-based processing, the two-step (sequential) deposition method^498^ would also apply to the vacuum-based process, with the organic precursor material(s) deposited after the inorganic(s). Moreover, the hybrid evaporation-solution method^499−501^ would allow the ready addition of various additives into the perovskite films while avoiding the usage of toxic solvents, such as the extensively employed amides and ureas (Figure 13a-ii). In this method, the inorganics are deposited first by the solvent-free vacuum-based deposition method. Then the organic precursor material(s), together with some organic additives, are processed with an environmentally friendly solvent, such as isopropanol. Thus, this will also overcome the issue of dissolving the underlying perovskite films in the tandem cell fabrication procedures, as it will only rely on the alcohol-based solvent(s), while the usage of polar solvents, DMF and DMSO, will be unnecessary. The application of these methods, however, is yet to be widely examined for both the single-junction mixed Sn−Pb PSCs and the corresponding all-perovskite tandems.

Blade coating is another promising deposition technique, which was successfully employed recently^15,32^ to fabricate highly efficient monolithic all-perovskite tandems, where both subcells were fabricated through this method (Figure 13a-iii, iv). Previous attempts mainly focused on fabricating the WBG subcells through blade coating, while still making use of spin coating for the thicker, more challenging to produce NBG subcells. One alternative sustainable processing route would be the adaptation of the hybrid evaporation-solution method, with the inorganics processed through the vacuum-based method while depositing the organic materials with solution-based blade coating.

Unlike small-area PSCs, perovskite solar modules require a three-step laser or mechanical scribing (namely, P1, P2, and P3) to connect the subcells in series.^32,502−505^ Therefore, besides advancing the large-area deposition methods, the modularization of all-perovskite tandems requires more technological advancements toward better design of the module structures and fabrication controls for minimizing the cell-to-module efficiency and stability gap. For example, the material interdiffusion between subcells and the reaction of halides and metal electrodes at the interconnecting areas of the modules has been a long-standing issue that raises severe loss of the device’s stability.^32,506^ Although some chemically inactive barrier materials can, to some extent, suppress the material interdiffusion in the module,^32,506^ it is generally accompanied by a reduction in the geometric FF of the modules due to the increased dead area. In general, the studies on the modularization of all-perovskite tandems are very limited,^15,32,444,507^ with the leading PCEs of no higher than 24%, lagging far behind the lab-based small-area cells. This suggests the pressing necessity for the further development of both large-area deposition methods and modularization technologies. We thus expect more efforts from both the academic and industrial communities regarding the modularization of the all-perovskite tandem PVs in the near future.

#### 4.4.2 Bifacial Devices

Bifacial devices are generally fabricated in such a configuration with the light that the light can reach the device from both sides to generate free charge carriers simultaneously in both subabsorbers (Figure 13b-i).^508^ This substrate cell structure would also be more realistic considering the installation ground,^509^ which generally reflects light. Thanks to the extra use of the reflected light, the bifacial tandems would generate more output power under the same irradiation compared to the monofacial counterparts and have mostly no current matching issue, especially when the ground has a high albedo.^464^ In addition, bifacial configurations would be industrially more promising with bifacial modules expected to account for a 55% share of the global PV market by 2031.^510^ The scientific research made on the development of bifacial all-perovskite tandems is very limited, however.

The tandems with substrate configuration can be fabricated through (i) the lamination of two single subcells or (ii) the sequential deposition of the layers stacked on. In the first method, two subcells with HTL and ETL layers are first fabricated separately with the glass-based substrate, and then, the subcells are mechanically laminated.^440^ For the second method, which is also the currently most employed method, all the layers with different functions are deposited sequentially with the transparent electrode ending on the top.^511^

The bifacial-orientated tandems can be fabricated beginning with either the WBG or the NBG subcells.^442^ Meanwhile, the substrate-oriented devices could potentially be fabricated on a large variety of opaque and inexpensive substrates,^70^ such as plastic and glass foils, for building/vehicle-integrated PVs.^512^ Highly flexible deposition routes would allow more freedom in the device design, thus likely reducing the fabrication cost of the solar panels further.

According to the limited attempts made so far, the best equivalent efficiency reported for the bifacial two-terminal double-junction all-perovskite tandems has reached 29.3% (with 30% of albedo light) (Figure 13b-ii),^42^ which is higher than the highest result published in the literature with the superstrate-oriented monofacial device, 28.5%.^77^ This suggests the exceptionally high potential of the bifacial tandem devices. From the stability aspect, the substrate-oriented tandems also outperform the superstrate analogues under both inert and ambient conditions (Figure 13b-iii).^70^ In this structure, the interconnecting and WBG perovskite layers are deposited sequentially above the NBG mixed Sn−Pb perovskite films. In this sense, the reactive species, such as the metal from the interconnecting layer and the halides from the WBG neat Pb perovskite films, will hardly reach the mixed Sn−Pb perovskite films underneath thanks to the protection by the ALD-SnO_2_ layer deposited above. Accordingly, the unencapsulated tandem devices can operate even over 250 h with no efficiency loss, substantially greater than the superstrate-oriented tandems tested under the same condition, i.e., with almost no efficiency retained in 20 h of continuous operation.^70^ Moreover, the utilization of the reflected light would also allow us to reduce the bandgap of the WBG subcell while maintaining a good match of the current between two subcells,^42,464^ meaning a reduction of the Br content at the X-site of the composition. Accordingly, this will reduce the extent of the halide segregation that normally causes instability.^476^ The bandgap of the WBG subcell can be reduced from the commonly optimized 1.78 eV down to 1.65 eV by reducing the amount of Br ions at the X-site while providing the bifacial cells with considerably enhanced performance.^42^ As demonstrated by these pioneering works, we think the community should pour more effort into the development of all-perovskite tandems with substrate configurations that are applicable for bifacial operation and, meanwhile, should also build a thorough reporting standard that would allow comparisons of the cell efficiencies.

#### 4.4.3 Flexible Devices

Besides rigid PSCs, flexible devices, both single junctions as well as multijunctions, have interesting applications, for example, in aerospace, vehicle-integrated photovoltaics, and wearable electronics.^512^ Furthermore, they would allow roll-to-roll processing, enabling efficient scale-up, as well as easy installation of the modules with decreased package weight.^513^ Although processing the NBG perovskite on flexible substrates comes with additional challenges,^514,515^ most research efforts in the direction of flexible all-perovskite tandem devices focus on resolving issues with the WBG subcells and the interconnect.^84,90,516^ This thus calls for further investigation into the fabrication of high-quality flexible NBG perovskite films in the community (Figure 13c).^81^

#### 4.4.4 Triple-Junction Tandems

With the development of all-perovskite tandems, multijunctions with more than two junctions are attracting more and more attention (Figure 13d-i).^240,517^ In principle, the higher energy yield can be readily realized by increasing the absorbers stacked in the tandems, if optical losses related to charge extraction and interconnecting difficulties can be overcome. Every additional junction will introduce optical losses due to the requirement to enable electron−hole recombination between every subcell. As demonstrated with extremely high EQE spectra for Si subcells in perovskite-on-silicon tandems, however, the optical losses with each additional subcell should be able to be mitigated to around 1%, with the appropriate optical design and management. Therefore, ultimately multijunction perovskites with four or five junctions should prove to deliver the highest efficiency and highest energy yields.^39^ It is our view, however, that for terrestrial applications surpassing three junctions will be unlikely, but this does give the realistic opportunity to realize close to 40% efficient PV cells.^24^ Importantly, when scaling up from two to three junctions, the NBG subcell does not need to be altered much, and insights into processing can be directly transferred from NBG subcells into multijunction tandems (Figure 13d-ii, iii, iv). The main challenge currently lies with the development of efficient front WBG subcells suitable for triple junctions, which implies bandgaps of over 1.9 eV.^93,100,101^ Although this is an important area for future research, this goes beyond the scope of the current Review, and we refer the reader to recent publications of PSCs with bandgaps of over 1.9 eV and triple-junction tandems.^93,100,101,518^ In addition, we expect more advancements in perovskite materials with an even wider bandgap to promise the success of tandem solar cells with the junction over three, e.g., quadruple-junction devices.

### 4.5 Section Summary

Overall, all-perovskite tandem solar cells have a huge potential for efficient and cheap photovoltaic applications in all kinds of environments. The possibility to tune perovskite film properties through its composition allows for maximizing power generation by combining a WBG and an NBG material in a tandem junction. However, the current performance of these devices is still far from their maximum potential. The main challenges and the strategies with the strongest potential to overcome them are as follows. (i) Current interconnecting layers have parasitic absorption, potentially react with perovskite materials and have a fragile fabrication process. The development of new interconnecting layers and tighter control of the deposition process would reduce the contact resistance, increase its transparency and avoid unwanted chemical reactions at the interfaces. (ii) Fully optimizing the current matching of the two WBG and NBG absorbers requires precise control of the bandgaps and thicknesses to ensure capturing all photons of specific energies. However, the high Br- content in WBG and Sn(II) content in NBG perovskites introduce further difficulties regarding their crystallization into compact and highly crystalline films. In particular, the insertion of Sn(II) in the structure strongly accelerates the crystallization process of the perovskite, making it hard to produce homogeneous and thick (>1000 nm) mixed Sn−Pb perovskite films. To this end, a deeper knowledge of Sn-containing perovskite crystallization is required, in order to develop novel strategies that can ensure high-quality thin film production (see section 2.1). In addition, conventional ETMs and HTMs absorb part of the spectrum, reducing the total amount of light that can be captured by the perovskite absorbers. We highlight the potential of SAM-modified contacts to avoid this problem and enhance device efficiency. (iii) Achieving long-term stability is a must in order to commercialize this type of device. The current obstacles for all-perovskite tandems in this direction are mostly linked to the NBG absorber. Mixed Sn−Pb perovskite devices normally suffer failure from loss of their MA^+^ and Sn(II) content; in addition, the acidic PEDOT:PSS and delaminating C_60_ layer make these compositions and cells even more unstable. Thus, we anticipate the future potential of MA-free mixed Sn−Pb perovskites for NBG absorbers stacked with optimal charge selective SAMs, in combination with advanced encapsulation techniques for the protection of the material against environmental factors. (iv) Thermal evaporation of the whole tandem fabrication would be desired for its upscaling. However, it is hard to obtain layers of the same quality as with solution-based methods. Here, the optimization of thermal evaporation processes, together with the development of hybrid fabrication methods, i.e., combining thermal evaporation- and solution-based methods, would help maintain a high film quality while advancing toward upscaling. (v) Finally, bifacial, flexible, and triple-junction devices still need to be developed to enable the full applicability of all-perovskite tandem solar cells.

## 5 Summary and Future Outlook

### 5.1 Summary

The performance of perovskite optoelectronic devices is highly affected by the nature of the different surfaces present in perovskite films and devices. Grain boundaries and interfaces of low quality will inevitably increase nonradiative recombination and hinder charge transport and extraction. For the particular case of Sn-containing perovskites, such as mixed Sn−Pb, surfaces are of exceptional concern. Sn-based perovskites suffer from higher defect densities, due to challenges with controlling crystallization and the tendency of Sn(II) to oxidize. In addition, mass loss mechanisms and ion movement phenomena in these Sn−Pb perovskites further complicate their development. Nevertheless, mixed Sn−Pb perovskites hold formidable potential for applications in both single-junction and tandem solar cells.

Reports in the literature present different methods to address the fragile surfaces in Sn-containing perovskites. On the one hand, controlling the crystallization process and preventing the oxidation of Sn(II) in the material allows for the fabrication of thin films with higher intrinsic quality and lower defect densities. On the other hand, different additives and treatments can be applied for the passivation of imperfect surfaces. Fullerene derivatives, Lewis bases, and ammonium species, properly functionalized to control their properties, are so far the most successful surface modification agents. While similar strategies have been successfully applied for neat Pb perovskites, Sn-containing perovskite films can particularly benefit from surface passivation strategies, considering their particularly defect-rich and imperfect character. The implementation of these surface treatments has allowed the community to quickly improve the efficiency and stability of mixed Sn−Pb PSCs.

Mixed Sn−Pb perovskites, when properly designed and processed, have shown excellent performance when implemented in all-perovskite tandem solar cells as the NBG rear absorber. However, fabricating a good all-perovskite tandem requires further efforts than obtaining a high-quality NBG. In particular, we should pursue good current matching between the NBG and the WBG to maximize the current generation, a good interconnecting layer to allow charges to recombine efficiently while maximizing forward transmission of light into the NBG absorber, a proper cell design that avoids degradation processes, and further development of the fabrication protocols and device structures. Reaching these objectives would enhance the future applicability and commercialization of all-perovskite tandem solar cells.

### 5.2 Future Outlook

While excellent efficiencies of over 23% and 29% have been achieved for mixed Sn−Pb single-junction PSCs and all-perovskite tandems, respectively, there is still a lot of progress to be made in terms of reproducibility, compositional and device structure engineering, and device stability. We underline the urgency for developing novel, simple processing protocols that reduce the number of defects and mobile ions generated in the perovskite films. Tracking the crystallization dynamics and understanding how the solvents or potential additives affect the perovskite colloids and the crystallization process will be a critical step forward. In particular, future studies should unravel the different crystallization dynamics between Sn and Pb, and how they are involved in the generation of metal distribution heterogeneities in the final perovskite films. In particular, techniques that can distinguish between Sn and Pb, such as hyperspectral imaging and nanofocused WAXS, can be excellent tools for evaluating the homogeneity of the mixed Sn−Pb films.

Tracking the generation and evolution of defect and mobile ion densities as well as understanding oxidation processes better may reveal new ways to mitigate ion migration and other degradations. The community should seek new methods to identify the defects and understand the characteristics and differences between them. Furthermore, new materials and methods should be developed for robust surface defect passivation. In particular, new studies should focus on the strengthening of the Sn−I bond, for instance, by altering the chemical environment or introducing new anions to bind Sn strongly at the weak points, such as surfaces. Alternatively, developing single-crystal PSCs would greatly reduce the defect density in the perovskite films and strengthen the robustness against degradation.

Finally, the community should bridge the gap between lab-scale research and commercialization of mixed Sn−Pb perovskite-based optoelectronic devices via, for example, vacuum-based thermal evaporation and green solvent-based, two-step hybrid evaporation-solution deposition assisted by blade coating. To improve the current matching and durability of the NBG absorber materials, we highlight the need for tighter control of the crystallization and new perovskite and device compositions with higher durability even under extreme conditions, for example, largely elevated temperatures and severe thermal cycles to match the practical application of this PV technology. If the mentioned issues can be resolved, we expect a big potential for perovskites on optimal substrates (e.g., rationally designed SAMs), combined with robust sealing techniques. Moving away from spin coating and toxic solvents toward more scalable deposition methods is hugely important. For instance, improved thermal evaporation processes and the development of novel hybrid deposition protocols will be beneficial for upscaling. In addition, a better understanding of the performance under mechanical stress will allow the development of flexible single-, double-, and multi-junction PVs. Finally, machine learning can aid the data-driven design of high-performance perovskite materials, which would ultimately accelerate the industrialization of the all-perovskite tandem PVs.
